# Supplementary material for: Plant carbonic anhydrase-like enzymes in neuroactive alkaloid biosynthesis
Source: Nature. 2023 Nov 8;624(7990):182–91. doi: 10.1038/s41586-023-06716-y (PMC10700139; doi:10.1038/s41586-023-06716-y)
Supplement: Supplementary file 1 — Supplementary Methods, Results, Figs. 1–23 and References. [file 41586_2023_6716_MOESM1_ESM.pdf]

nature portfolio

https://doi.org/10.1038/s41586-023-06716-y

Supplementary information

Plant carbonic anhydrase-like enzymes in neuroactive alkaloid biosynthesis

In the format provided by the authors and unedited

SUPPLEMENTARY INFORMATION

Plant carbonic anhydrase-like enzymes in neuroactive alkaloid biosynthesis

Ryan S. Nett,[1,2,5]* Yaereen Dho,[3] Chun Tsai,[2] Daria Passow,[4] Jaime Martinez Grundman,[5] Yun-Yee Low,[6] & Elizabeth S. Sattely[1,2]*

[1]Department of Chemical Engineering, Stanford University, Stanford, CA 94305
[2]HHMI, Stanford University, Stanford, CA 94305
[3]Department of Chemistry, Stanford University, Stanford, CA 94305
[4]Biophysics Program, Stanford University, Stanford, CA 94305
[5]Department of Molecular and Cellular Biology, Harvard University, Cambridge, MA 02138
[6]Department of Chemistry, Faculty of Science, Universiti Malaya, 50603 Kuala Lumpur, Malaysia
*co-corresponding authors

	page(s)
**Supplementary Methods**	2-3
**Supplementary Results**	4
**Supplementary Figures**	5-27
Figure 1. Background on the chemistry and biogenesis of Lycopodium alkaloids.	5-6
Figure 2. Phylogenetic analysis of CAH family proteins across multiple kingdoms of life.	7
Figure 3. Heterodimer modeling for *Pt*CAL-1 and *Pt*CAL-2.	8
Figure 4. Verification and additional characterization of *Pt*2OGD-3 function.	9-10
Figure 5. Verification and additional characterization of function for *Pt*2OGD-1 and *Pt*2OGD-2.	11-12
Figure 6. Detection of early biosynthetic intermediates in extracts of *P. tetrastichus*.	12
Figure 7. Detection of downstream biosynthetic intermediates in extracts of *P. tetrastichus.*	13
Figure 8. ^{1}H NMR spectrum from the synthesis of **6** stereoisomers.	14
Figure 9. ^{13}C NMR spectrum from the synthesis of **6** stereoisomers.	15
Figure 10. ^{1}H NMR (crude) of the purified product (putative **9**, *m/z* 247.2169) of *Pt*CAL-1/*Pt*CAL-2.	16
Figure 11. ^{1}H NMR (crude) of putative **9** (zoomed in).	17
Figure 12. NMR assignment of the oxidized scaffold by-product **9'** (*m/z* 263.2118).	18
Figure 13. ^{1}H NMR spectrum of the oxidized scaffold by-product **9'** (*m/z* 263.2118).	19
Figure 14. ^{13}C NMR spectrum of the oxidized scaffold by-product **9'** (*m/z* 263.2118).	20
Figure 15. COSY spectrum of the oxidized scaffold by-product **9'** (*m/z* 263.2118).	21
Figure 16. HMBC spectrum of the oxidized scaffold by-product **9'** (*m/z* 263.2118).	22
Figure 17. HSQC spectrum of the oxidized scaffold by-product **9'** (*m/z* 263.2118).	23
Figure 18. TOCSY spectrum of the oxidized scaffold by-product **9'** (*m/z* 263.2118).	24
Figure 19. ^{1}H NMR spectrum ($CDCl_3$, 400 MHz) of flabellidine (**10**) isolated from *Lycopodium platyrhizoma*.[1]	25
Figure 20. ^{13}C NMR spectrum ($CDCl_3$, 100 MHz) of flabellidine (**10**) isolated from *Lycopodium platyrhizoma*.[1]	25
Figure 21. ^{1}H NMR spectrum ($CDCl_3$, 400 MHz) of casuarinine H (**20**) isolated from *Lycopodium platyrhizoma*.[1]	26
Figure 22. ^{13}C NMR spectrum ($CDCl_3$, 100 MHz) of casuarinine H (**20**) isolated from *Lycopodium platyrhizoma*.[1]	27
Figure 23. Raw images for Western blots.	28
**Supplementary References**	29

SUPPLEMENTARY METHODS

HPLC method parameters

**Instrument:** 6520 LC-MS	
**Method:** HILIC 17-min gradient	
**Column:** Poroshell 120 HILIC-Z column (Agilent, 2.7 µm, 2.1 × 100 mm)	
**Solvent A:** water with 0.1% (v/v) formic acid, 10 mM ammonium formate	
**Solvent B:** 9:1 ACN:water with 0.1% (v/v) formic acid, 10 mM ammonium formate	
**Flow rate:** 0.25 mL/min	
**Injection volume:** 2 uL	
**Notes:** Used for analyzing early biosynthetic intermediates (**1** thru **9**)	
**% Solvent B**	**time (min)**
100	0
100	3
60	8
100	9
100	17

**Instrument:** 6546 LC-MS	
**Method:** C18 9-min gradient	
**Column:** ZORBAX RRHD Eclipse Plus C18 column (Agilent, 1.8 µm, 2.1 x 50 mm)	
**Solvent A:** water with 0.1% (v/v) formic acid	
**Solvent B:** ACN with 0.1% (v/v) formic acid	
**Flow rate:** 0.6 mL/min	
**Injection volume:** 1 uL	
**Notes:** Used for analyzing downstream biosynthetic intermediates (**10** thru **24**)	
**% Solvent B**	**time (min)**
3	0
3	0.50
50	6.50
95	6.51
95	8.00
3	8.01
3	9.00

**Instrument:** 6520 LC-MS	
**Method:** Chiral 33-min gradient	
**Column:** CHIRALPAK® IC-3 column (Daicel, 3 µm, 4.6 x 100 mm)	
**Solvent A:** water with 0.1% (v/v) formic acid	
**Solvent B:** ACN with 0.1% (v/v) formic acid	
**Flow rate:** 0.4 mL/min	
**Injection volume:** 2 uL	
**Notes:** Used for analyzing *N*-acetylated stereoisomers of **4**, **5**, and **6**.	
**% Solvent B**	**time (min)**
3	0
3	1
70	21
97	22
97	27
3	28
3	33

Mass spectrometer parameters

**Instrument:** 6520 LC-MS	
**Mode**	ESI positive, MS
**Drying gas temp**	300 ºC
**Drying gas flow rate**	11 L/min
**Nebulizer**	35 psig
**Fragmentor**	150 V
**Skimmer**	65 V
**OCT 1 Rf Vpp**	750 V
**Vcap**	3500 V

**Instrument:** 6546 LC-MS	
**Mode**	ESI positive, MS
**Drying gas temp**	325 ºC
**Drying gas flow rate**	10 L/min
**Nebulizer**	35 psig
**Fragmentor**	135 V
**Skimmer**	45 V
**OCT 1 Rf Vpp**	750 V
**Vcap**	4000 V

SUPPLEMENTARY RESULTS

Identification of downstream oxidases

The most highly reduced Lycopodium alkaloid with the same core "lycodane" scaffold as **17** is flabellidine (**10**),[2] which contains an *N*-acetyl group on the A ring nitrogen. This molecule had previously been isolated from plants that also produce more highly oxidized lycodane-type alkaloids,[1] suggesting it to be a logical precursor, and we used this for substrate co-infiltration into *N. benthamiana* leaves expressing our oxidase gene candidates. Through this approach, we identified a pair of 2OGD enzymes that act to consume **10** and produce oxidized products. The first of these enzymes (*Pt*2OGD-4) appeared to oxidize **10** to a molecule with an exact mass that is consistent with the installation of a carbonyl (proposed structure **11,** $[M+H]^+$ = *m/z* 303.2067) (**Extended Data Fig 7**). Additionally, we observed minor products that corresponded to the addition of a hydroxyl group ($[M+H]^+$ = *m/z* 305.2224). We speculate that this may represent a hemiaminal intermediate en route to the carbonyl, that could isomerize to form different scaffold types such as that of the lycopodine-related alkaloids (**Fig 1a, Extended Data Fig 7**). The second enzyme (*Pt*2OGD-5) consumed **10** and produced a new compound with an exact mass indicative of a desaturation (proposed structure **12**, $[M+H]^+$ = *m/z* 287.2118) (**Extended Data Fig 8**). In agreement with these observed activities, transient co-expression of *Pt*2OGD-4 and *Pt*2OGD-5 with **10** as substrate led to the formation of a compound with both the carbonyl and the desaturation (proposed structure **13**, $[M+H]^+$ = *m/z* 301.1911) (**Extended Data Fig 8**). Though both *Pt*2OGD-4 and *Pt*2OGD-5 could act on **10**, we observed greater consumption of **10** by *Pt*2OGD-4 than *Pt*2OGD-5 (~6-fold vs. ~3-fold depletion of **10**, respectively, P = 0.13) in multiple experiments (**Extended Data Fig 8e**). This suggested that the major order of reactions is *Pt*2OGD-4 oxidation of **10** to produce **11**, followed by desaturation catalyzed by *Pt*2OGD-5 to produce **13**. However, the opposite order of events could also potentially occur in the native plant. Without authentic standards for these substrates, it was not initially possible to confirm the location of these oxidations, but we suspected that *Pt*2OGD-4 was catalyzing formation of the carbonyl to yield the ring A lactam, while *Pt*2OGD-5 was installing the 8,15-double bond. Eventually, we determined that **13** was converted into lycophlegmarinine D (**14**) via the *N*-deacetylation activity of *Pt*ABH-1 (**Extended Data Fig 9**). Because we could confirm the structure of **14**, this verified the proposed activities of *Pt*2OGD-4 and *Pt*2OGD-5 for the installation of the A-ring carbonyl and the 8,15-double bond, respectively.

Characterization of a Lycopodium alkaloid metabolic network

Once we had established a biosynthetic pathway from **10** to **17**, we next wanted to determine whether we could access other previously reported Lycopodium alkaloids that appear to differ from **17** pathway intermediates only in their degree of unsaturation. To assess the potential production of these molecules, we reconstituted biosynthesis from **10** with specific 2OGD desaturases (*Pt*2OGD-3 and/or *Pt*2OGD-5) omitted from the co-expressed set of enzymes. We found that omission of *Pt*2OGD-5 led to the consecutive production of the following 8,15-dihydro compounds: **18**, **19**, casuarinine H (**20**), and 8,15-dihydrohuperzine A (**21**), which were all confirmed via authentic standards (**Extended Data Fig 10b & Supplementary Fig. 5**). In a similar fashion, the omission of *Pt*2OGD-3 led to the production of putative 2,3-dihydro congeners: 2,3-dihydrohuperzine C (proposed structure **22**, $[M+H]^+$ = *m/z* 245.1658, $[M-NH_2]^+$ = *m/z* 228.1383) and 2,3-dihydrohuperzine A (proposed structure **23**, $[M+H]^+$ = *m/z* 245.1658, $[M-NH_2]^+$ = *m/z* 228.1383), which to our knowledge have not previously been described (**Extended Data Fig 10c & Supplementary Fig. 5**). Finally, omission of both *Pt*2OGD-5 and *Pt*2OGD-3 led to the formation of lycoplatyrine B (**24**) and a previously undescribed Lycopodium alkaloid that we predict to be 2,3,8,15-tetrahydrohuperzine A (proposed structure **25**, $[M+H]^+$ = *m/z* 247.1805, $[M-NH_2]^+$ = *m/z* 230.1539) (**Extended Data Fig 10d & Supplementary Fig. 5**). These results confirmed the relative promiscuity of these downstream enzymes, thereby allowing for the production of a network of Lycopodium alkaloids.

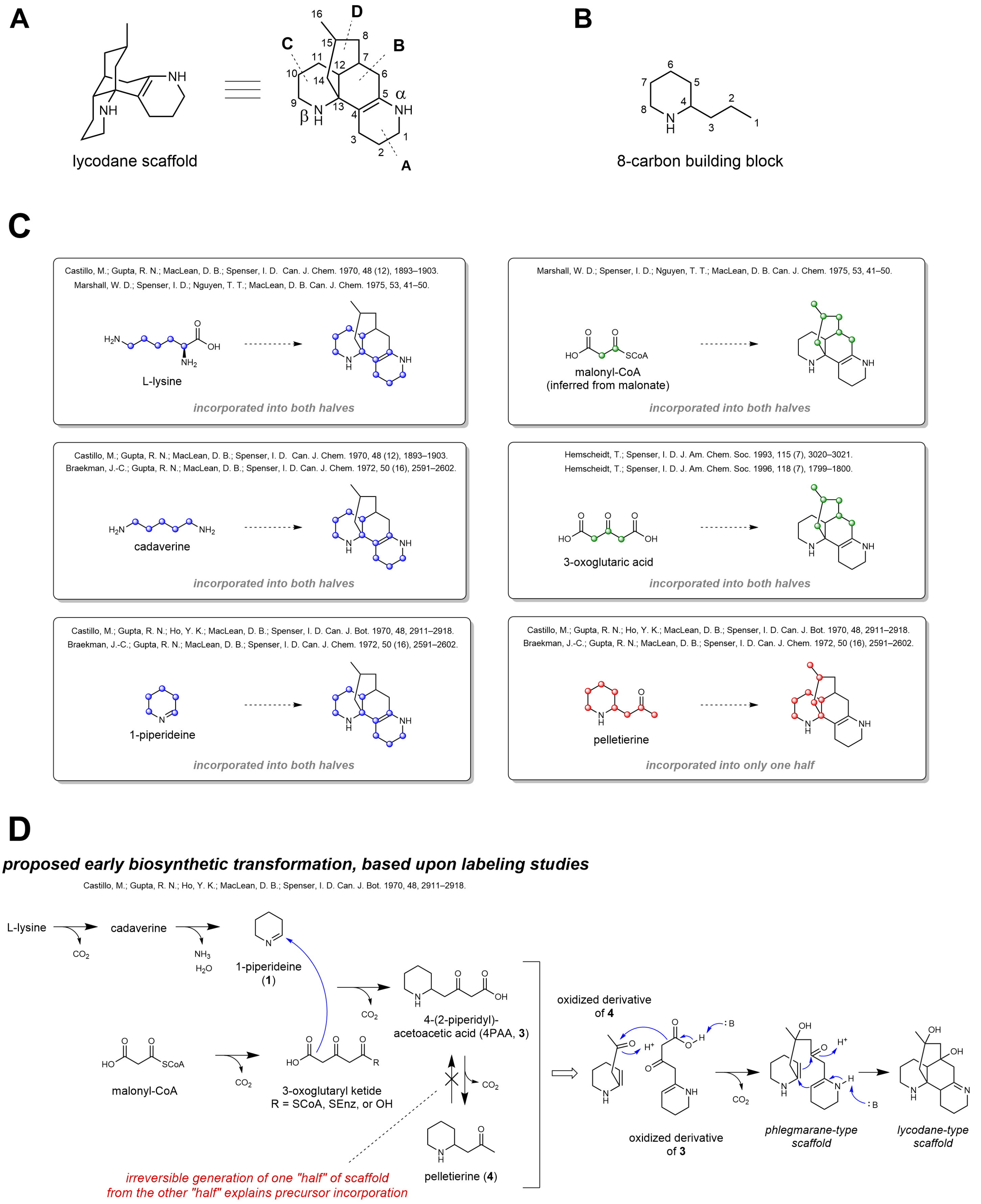

Figure 1. Background on the chemistry and biogenesis of Lycopodium alkaloids. A) Chemical schematic of the lycodane scaffold, a major structural class of Lycopodium alkaloid, in two different orientations. Also shown is atom numbering and ring annotation for this scaffold. Note that this is not an observed alkaloid, but a representation of the simplest

form of this scaffold. **B)** Chemical schematic and carbon numbering of the 8-carbon Lycopodium alkaloid "building block", which is representative of pelletierine (**4**) and some downstream intermediates. As above, this structure is only a representation of the carbon skeleton, and has not been observed as a native Lycopodium alkaloid. **C)** Summary of previous isotope labeling studies that have defined Lycopodium alkaloid precursors. Colored spheres are used to track location of carbons. Note that these do not represent the specific results of the prior labeling studies, but rather summarize the proposed incorporation of substrate based upon the collective data. Also, isotope labeling studies were performed to evaluate incorporation of substrates into lycopodine, which bears a different scaffold. The incorporation into the lycodane scaffold is inferred, and as previously been postulated.[3] Relevant manuscripts for each isotope labeling study are shown within the figure. **D)** Biosynthetic proposal[3] accounting for all of the isotope labeling studies. Note that molecules downstream of **3** and **4** have not been evaluated and are hypothetical.

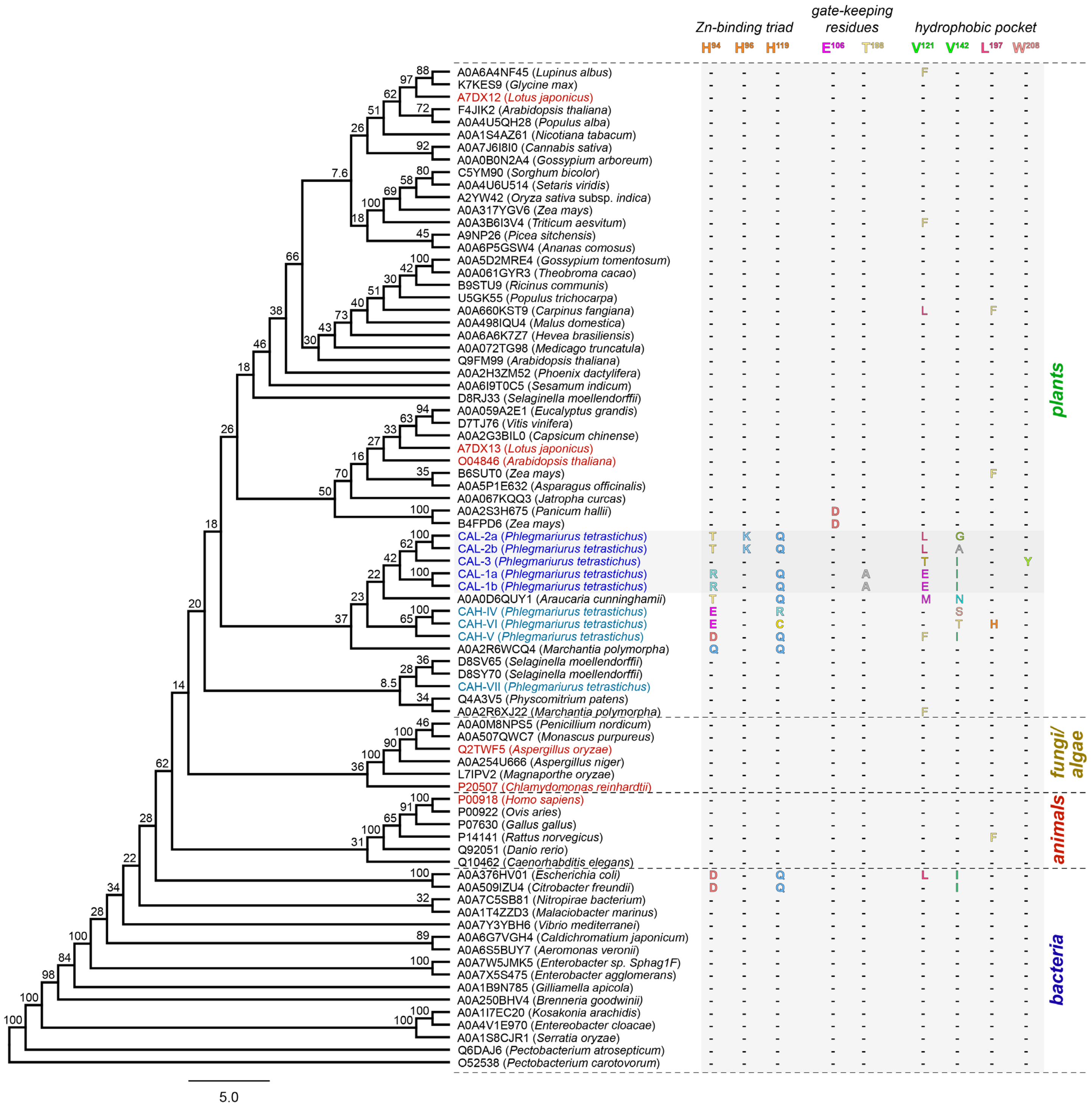

Figure 2. Phylogenetic analysis of CAH family proteins across multiple kingdoms of life. Sequences containing an alpha-carbonic anhydrase domain were downloaded from the UniProt database, aligned using MUSCLE, and a phylogenetic tree was generated in Geneious software using a Neighbor-Joining method. Bootstrap values (100 replicates) are shown at nodes. The scale bar indicates substitutions per amino acid Located next to the tree are the amino acid residues that pertain to the histidine-binding triad, gate keeping residues, and substrate binding pocket of canonical carbonic anhydrases. The human CA2 protein (UniProt ID: P00918) is used as the reference sequence, and reference amino acid number is based upon this protein. Consensus with this reference is shown as a dash (“ - ”), while changes are shown by listing the mutated amino acid. Biochemically verified CAH proteins with canonical activity are highlighted in red. The CAL proteins identified and characterized in this study are shown in blue and are highlighted with a gray box. Other CAH-like proteins identified within our *P. tetrastichus* transcriptome, but with unknown function, are shown in light blue. A selected subset of these sequences are shown within the phylogenetic tree in **Figure 4b**.

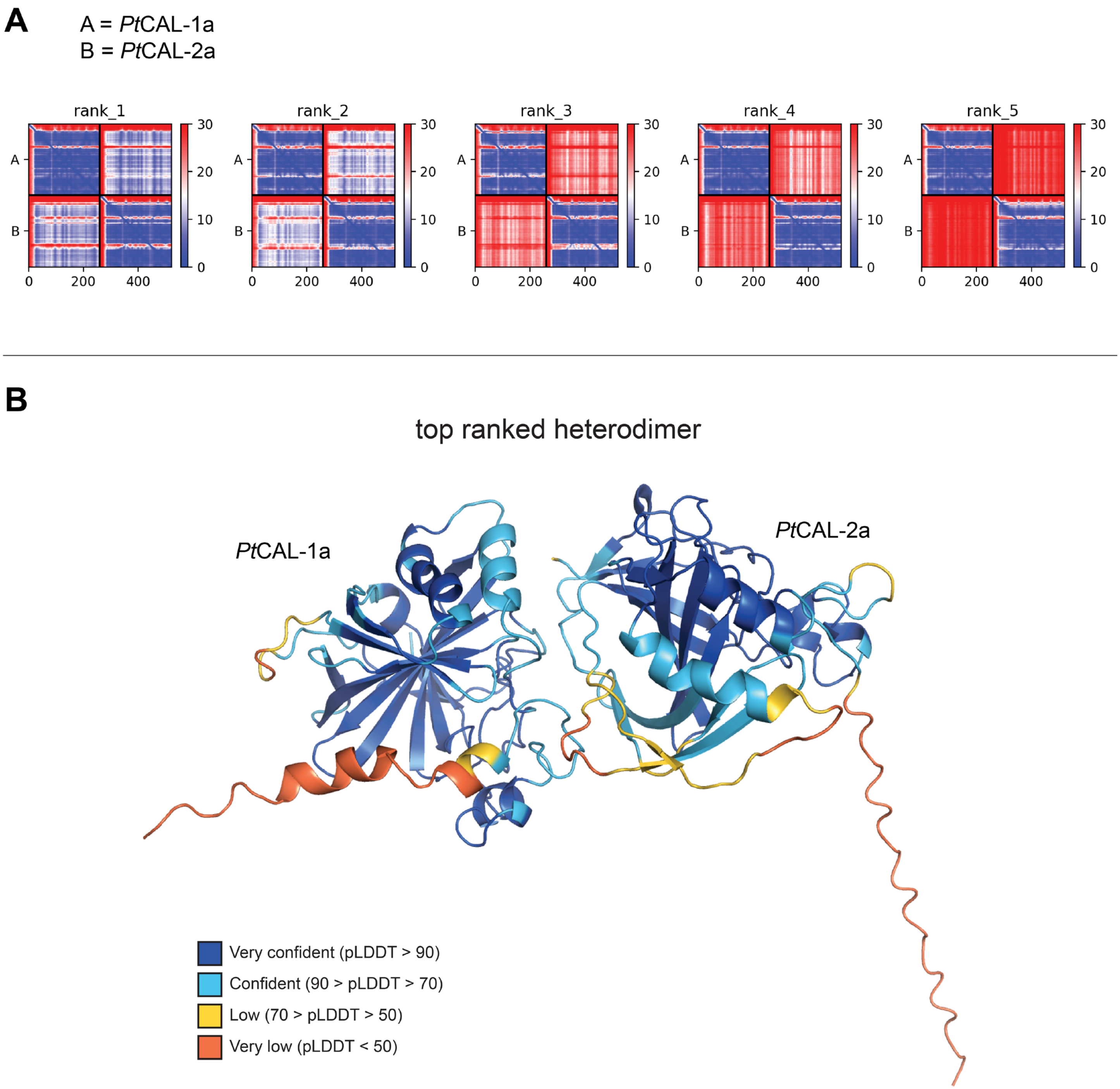

Figure 3. Heterodimer modeling for *Pt*CAL-1 and *Pt*CAL-2. The potential for heterodimerization between *Pt*CAL-1a and *Pt*CAL-2a was computationally assessed using AlphaFold-Multimer via ColabFold (v1.5.2). **A)** Predicted aligned error (PAE) plots for the top five ranked heterodimer complexes predicted by AlphaFold-Multimer. In this plots, chain "A" represents *Pt*CAL-1a and chain "B" represents *Pt*CAL-2a. Note that these models were generated using CAL proteins in which the predicted N-terminal signal peptide had been removed. The PAE plots display the error in Angstroms between each pair of amino acid residues. **B)** Structural prediction of the top ranked heterodimer for *Pt*CAL-1a and *Pt*CAL-2, with each protein colored according to the pLDDT confidence score per residue.

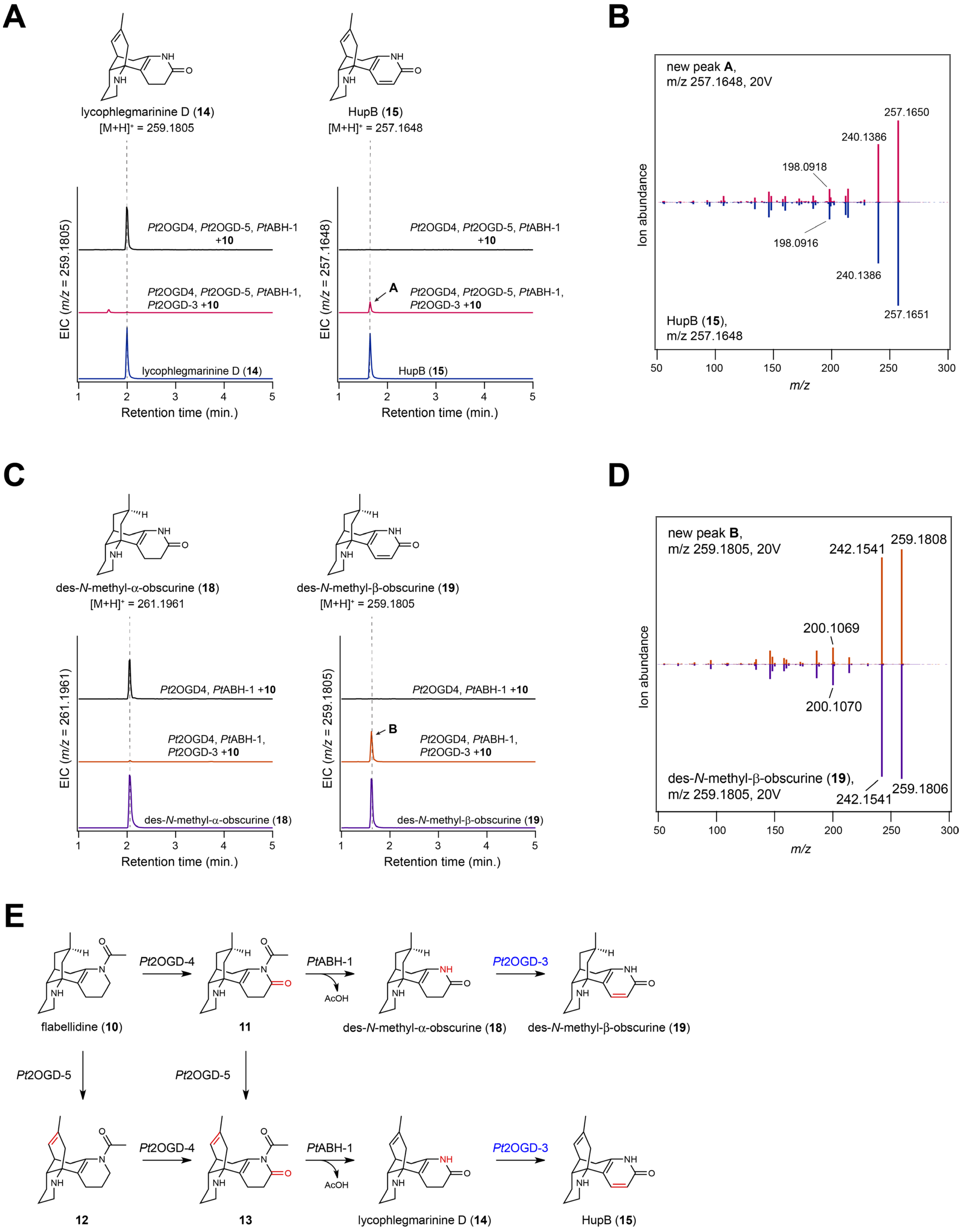
A
lycophlegmarinine D (14)
[M+H]+ = 259.1805
HupB (15)
[M+H]+ = 257.1648
EIC (m/z = 259.1805)
EIC (m/z = 257.1648)
Pt2OGD4, Pt2OGD-5, PtABH-1 +10
Pt2OGD4, Pt2OGD-5, PtABH-1, Pt2OGD-3 +10
lycophlegmarinine D (14)
HupB (15)
Retention time (min.)
B
new peak A, m/z 257.1648, 20V
257.1650
240.1386
198.0918
198.0916
240.1386
257.1651
HupB (15), m/z 257.1648
Ion abundance
m/z
C
des-N-methyl-α-obscurine (18)
[M+H]+ = 261.1961
des-N-methyl-β-obscurine (19)
[M+H]+ = 259.1805
EIC (m/z = 261.1961)
EIC (m/z = 259.1805)
Pt2OGD4, PtABH-1 +10
Pt2OGD4, PtABH-1, Pt2OGD-3 +10
des-N-methyl-α-obscurine (18)
des-N-methyl-β-obscurine (19)
Retention time (min.)
D
new peak B, m/z 259.1805, 20V
242.1541
259.1808
200.1069
200.1070
des-N-methyl-β-obscurine (19), m/z 259.1805, 20V
242.1541
259.1806
Ion abundance
m/z
E
flabellidine (10)
11
des-N-methyl-α-obscurine (18)
des-N-methyl-β-obscurine (19)
12
13
lycophlegmarinine D (14)
HupB (15)
Pt2OGD-4
PtABH-1
Pt2OGD-3
Pt2OGD-5
AcOH

Figure 4. Verification and additional characterization of *Pt*2OGD-3 function. A) Transient expression of *Pt*2OGD-3 with *Pt*2OGD-4, *Pt*2OGD-5, and *Pt*ABH-1 in *N. benthamiana* with co-infiltration of **10** as substrate. Shown are LC-MS extracted ion chromatograms (EICs) for the product of *Pt*2OGD-4/*Pt*2OGD-5/*Pt*ABH-1 (**14**, *m/z* 259.1805, left panel) and the product (A) of *Pt*2OGD-3 that corresponds to a desaturation ($[M+H]^+$ = *m/z* 257.1648, right panel), which is confirmed to be **15** via comparison to an authentic standard. **B)** MS/MS spectra of the new compound "A" (*m/z* 257.1648, 20V) in comparison to that of **15** (*m/z* 257.1648, 20V). **C)** Transient expression of *Pt*2OGD-3 with *Pt*2OGD-4 and *Pt*ABH-1 (*Pt*2OGD-5 omitted) in *N. benthamiana* with co-infiltration of **10** as substrate. Shown are LC-MS extracted ion chromatograms (EICs) for the product of *Pt*2OGD-4/*Pt*ABH-1 (**18**, *m/z* 261.1961, left panel) and a new product (B) of *Pt*2OGD-3 that corresponds to a desaturation, ($[M+H]^+$ = *m/z* 259.1805, right panel), which is confirmed to be **19** via comparison to an authentic standard. **D)** MS/MS spectra of the new compound "B" (*m/z* 259.1805, 20V) in comparison to that of **19** (*m/z* 259.1805). **E)** Biosynthetic proposal for the activity of *Pt*2OGD-3, which can act on either **18** or **14** to yield the A ring pyridone-containing structures of **19** or **15**, respectively. Note that we had previously demonstrated *Pt*2OGD-3 activity on **18** as a substrate.[4]

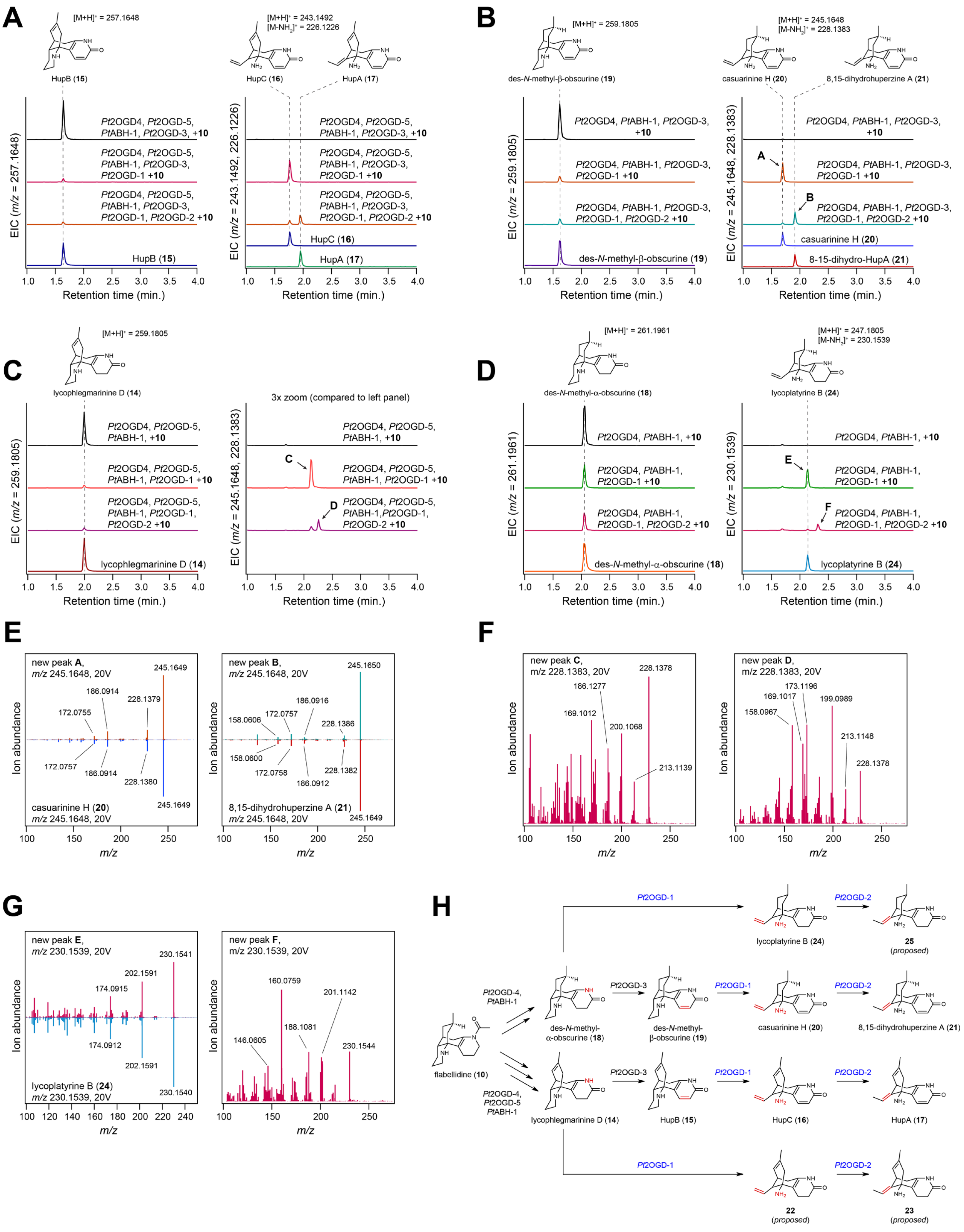
A
[M+H]+ = 257.1648
HupB (15)
EIC (m/z = 257.1648)
Pt2OGD4, Pt2OGD-5, PtABH-1, Pt2OGD-3, +10
Pt2OGD4, Pt2OGD-5, PtABH-1, Pt2OGD-3, Pt2OGD-1 +10
Pt2OGD4, Pt2OGD-5, PtABH-1, Pt2OGD-3, Pt2OGD-1, Pt2OGD-2 +10
HupB (15)
Retention time (min.)
[M+H]+ = 243.1492
[M-NH2]+ = 226.1226
HupC (16)
HupA (17)
EIC (m/z = 243.1492, 226.1226)
HupC (16)
HupA (17)
B
[M+H]+ = 259.1805
des-N-methyl-β-obscurine (19)
EIC (m/z = 259.1805)
Pt2OGD4, PtABH-1, Pt2OGD-3, +10
Pt2OGD4, PtABH-1, Pt2OGD-3, Pt2OGD-1 +10
Pt2OGD4, PtABH-1, Pt2OGD-3, Pt2OGD-1, Pt2OGD-2 +10
des-N-methyl-β-obscurine (19)
[M+H]+ = 245.1648
[M-NH2]+ = 228.1383
casuarinine H (20)
8,15-dihydrohuperzine A (21)
EIC (m/z = 245.1648, 228.1383)
A
B
casuarinine H (20)
8-15-dihydro-HupA (21)
C
[M+H]+ = 259.1805
lycophlegmarinine D (14)
EIC (m/z = 259.1805)
Pt2OGD4, Pt2OGD-5, PtABH-1, +10
Pt2OGD4, Pt2OGD-5, PtABH-1, Pt2OGD-1 +10
Pt2OGD4, Pt2OGD-5, PtABH-1, Pt2OGD-1, Pt2OGD-2 +10
lycophlegmarinine D (14)
3x zoom (compared to left panel)
EIC (m/z = 245.1648, 228.1383)
C
D
D
[M+H]+ = 261.1961
des-N-methyl-α-obscurine (18)
EIC (m/z = 261.1961)
Pt2OGD4, PtABH-1, +10
Pt2OGD4, PtABH-1, Pt2OGD-1 +10
Pt2OGD4, PtABH-1, Pt2OGD-1, Pt2OGD-2 +10
des-N-methyl-α-obscurine (18)
[M+H]+ = 247.1805
[M-NH2]+ = 230.1539
lycoplatyrine B (24)
EIC (m/z = 230.1539)
E
F
lycoplatyrine B (24)
E
new peak A, m/z 245.1648, 20V
casuarinine H (20) m/z 245.1648, 20V
new peak B, m/z 245.1648, 20V
8,15-dihydrohuperzine A (21) m/z 245.1648, 20V
Ion abundance
m/z
F
new peak C, m/z 228.1383, 20V
new peak D, m/z 228.1383, 20V
G
new peak E, m/z 230.1539, 20V
lycoplatyrine B (24) m/z 230.1539, 20V
new peak F, m/z 230.1539, 20V
H
Pt2OGD-1
Pt2OGD-2
lycoplatyrine B (24)
25 (proposed)
Pt2OGD-4, PtABH-1
des-N-methyl-α-obscurine (18)
Pt2OGD-3
des-N-methyl-β-obscurine (19)
casuarinine H (20)
8,15-dihydrohuperzine A (21)
flabellidine (10)
Pt2OGD-4, Pt2OGD-5 PtABH-1
lycophlegmarinine D (14)
HupB (15)
HupC (16)
HupA (17)
22 (proposed)
23 (proposed)

Figure 5. Verification and additional characterization of function for *Pt*2OGD-1 and *Pt*2OGD-2. A) Transient expression of *Pt*2OGD-1 and *Pt*2OGD-2 with *Pt*2OGD-4, *Pt*2OGD-5, *Pt*ABH-1, and *Pt*OGD-3 in *N. benthamiana* with co-infiltration of **10** as substrate. Shown are LC-MS extracted ion chromatograms (EICs) for the product of *Pt*2OGD-4/*Pt*2OGD-5/*Pt*ABH-1/*Pt*2OGD-3 (**15**, *m/z* 257.1648, left panel) and the products of *Pt*2OGD-1 and *Pt*2OGD-2 ($[M+H]^+$ = *m/z* 243.1492, $[M-NH_2]^+$ = 226.1226, right panel), which are confirmed to be **16** and **17** via comparisons to authentic standards. **B)** Transient expression of *Pt*2OGD-1 and *Pt*2OGD-2 with *Pt*2OGD-4, *Pt*ABH-1, and *Pt*2OGD-3 (*Pt*2OGD-5 omitted) in *N. benthamiana* with co-infiltration of **10** as substrate. Shown are LC-MS extracted ion chromatograms (EICs) for the product of *Pt*2OGD-4/*Pt*ABH-1/*Pt*2OGD-3 (**19**, *m/z* 259.1805, left panel) and two new products (A & B) from the activities of *Pt*2OGD-1 and *Pt*2OGD-2 ($[M+H]^+$ = *m/z* 245.1648, $[M-NH_2]^+$ = 228.1383 right panel), which are confirmed to be **20** and **21** via comparisons to authentic standards. **C)** Transient expression of *Pt*2OGD-1 and *Pt*2OGD-2 with *Pt*2OGD-4, *Pt*ABH-1, and *Pt*2OGD-5 (*Pt*2OGD-3 omitted) in *N. benthamiana* with co-infiltration of **10** as substrate. Shown are LC-MS extracted ion chromatograms (EICs) for the product of *Pt*2OGD-4/*Pt*ABH-1/*Pt*2OGD-5 (**14**, *m/z* 259.1805, left panel) and two new products (C & D) from the activities of *Pt*2OGD-1 and *Pt*2OGD-2 ($[M+H]^+$ = *m/z* 245.1648, $[M-NH_2]^+$ = 228.1383 right panel), that putative pertain to the 2,3-dihydro congeners of **16** and **17**. **D)** Transient expression of *Pt*2OGD-1 and *Pt*2OGD-2 with *Pt*2OGD-4 and *Pt*ABH-1 (*Pt*2OGD-3 and *Pt*2OGD-5 omitted) in *N. benthamiana* with co-infiltration of **10** as substrate. Shown are LC-MS extracted ion chromatograms (EICs) for the product of *Pt*2OGD-4/*Pt*ABH-1/ (**18**, *m/z* 261.1961, left panel) and two new products (E & F) from the activities of *Pt*2OGD-1 and *Pt*2OGD-2 ($[M+H]^+$ = *m/z* 247.1805, $[M-NH_2]^+$ = 230.1539 right panel). "E" is confirmed to be **24** via comparison to an authentic standard, and "F" is proposed to be the 2,3,8,15-tetrahydro congener of **17**. **E)** MS/MS spectra of the new compound "A" (*m/z* 245.1648, 20V) in comparison to that of **20** (*m/z* 245.1648, 20V) and "B" (*m/z* 245.1648, 20V) in comparison to **21** (*m/z* 245.1648, 20V). **F)** MS/MS spectra of the new compounds "C" and "D" (both *m/z* 228.1383, 20V). **G)** MS/MS spectra of the new compound "E" (*m/z* 230.1539, 20V) in comparison to that of **24** (*m/z* 230.1539, 20V), as well as that of "F" (*m/z* 230.1539. **H)** Biosynthetic proposal for the activities of *Pt*2OGD-1 and *Pt*2OGD-2. These enzymes appear to be able to act independently of the degree of unsaturation of precursors. Note that we had previously demonstrated *Pt*2OGD-1 and *Pt*2OGD-2 activity for conversion of **15** into **16** and **17**, as well as the conversion of **19** into **20** and **21**.[4] However, in this prior work, we were not able to confirm the nature of **20** and **21** with authentic standards, as shown here.

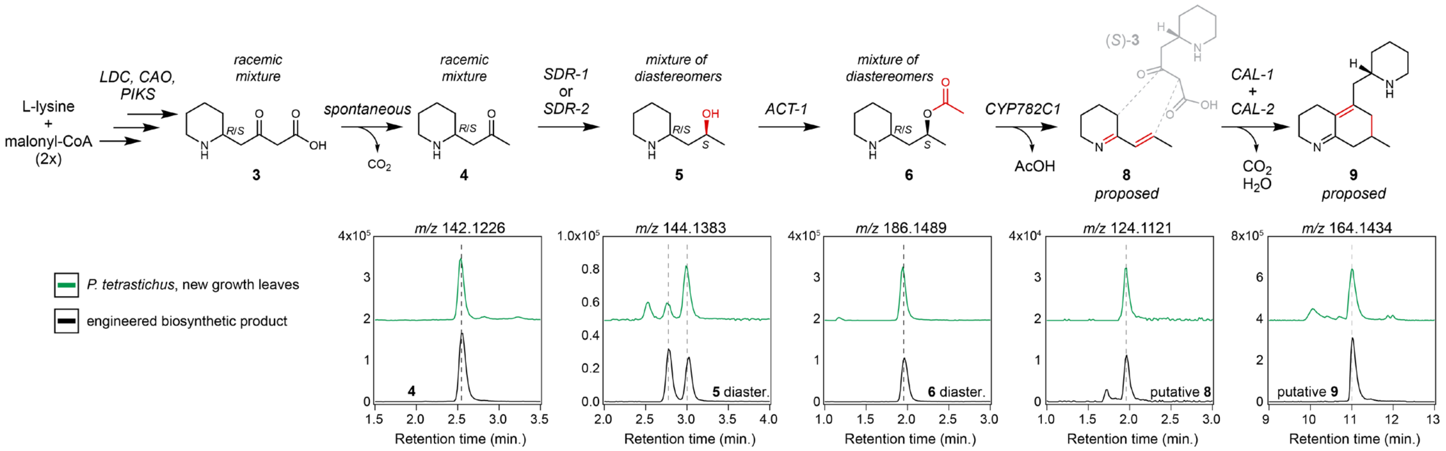

Figure 6. Detection of early biosynthetic intermediates in extracts of *P. tetrastichus*. Shown are LC-MS extracted ion chromatograms (EICs) for the various upstream intermediates of Lycopodium alkaloid biosynthesis from the extracts of the native plant (*P. tetrastichus*, green traces) and from the compounds generated through metabolic engineering in *N. benthamiana* (black traces). All samples here were analyzed with HILIC LC-MS. Note that diastereomers of **6** are not separated via HILIC analysis.

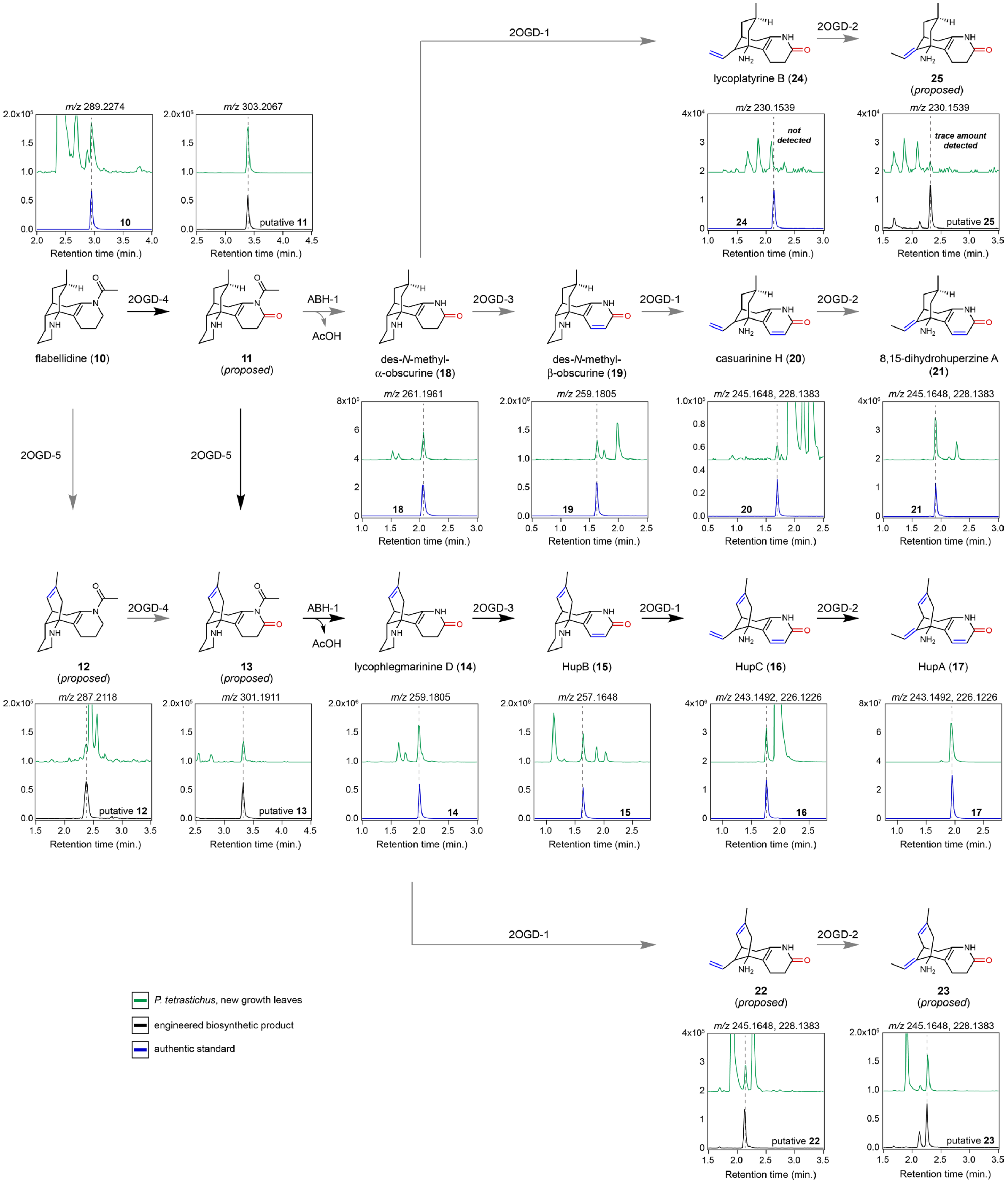

Figure 7. Detection of downstream biosynthetic intermediates in extracts of *P. tetrastichus*. Shown are LC-MS extracted ion chromatograms (EICs) for the downstream intermediates of Lycopodium alkaloid biosynthesis from the extracts of the native plant (*P. tetrastichus*, green traces), from the compounds generated through pathway engineering in *N. benthamiana* (black traces), and for authentic standards (blue), if available. All samples were analyzed via C18 LC-MS.

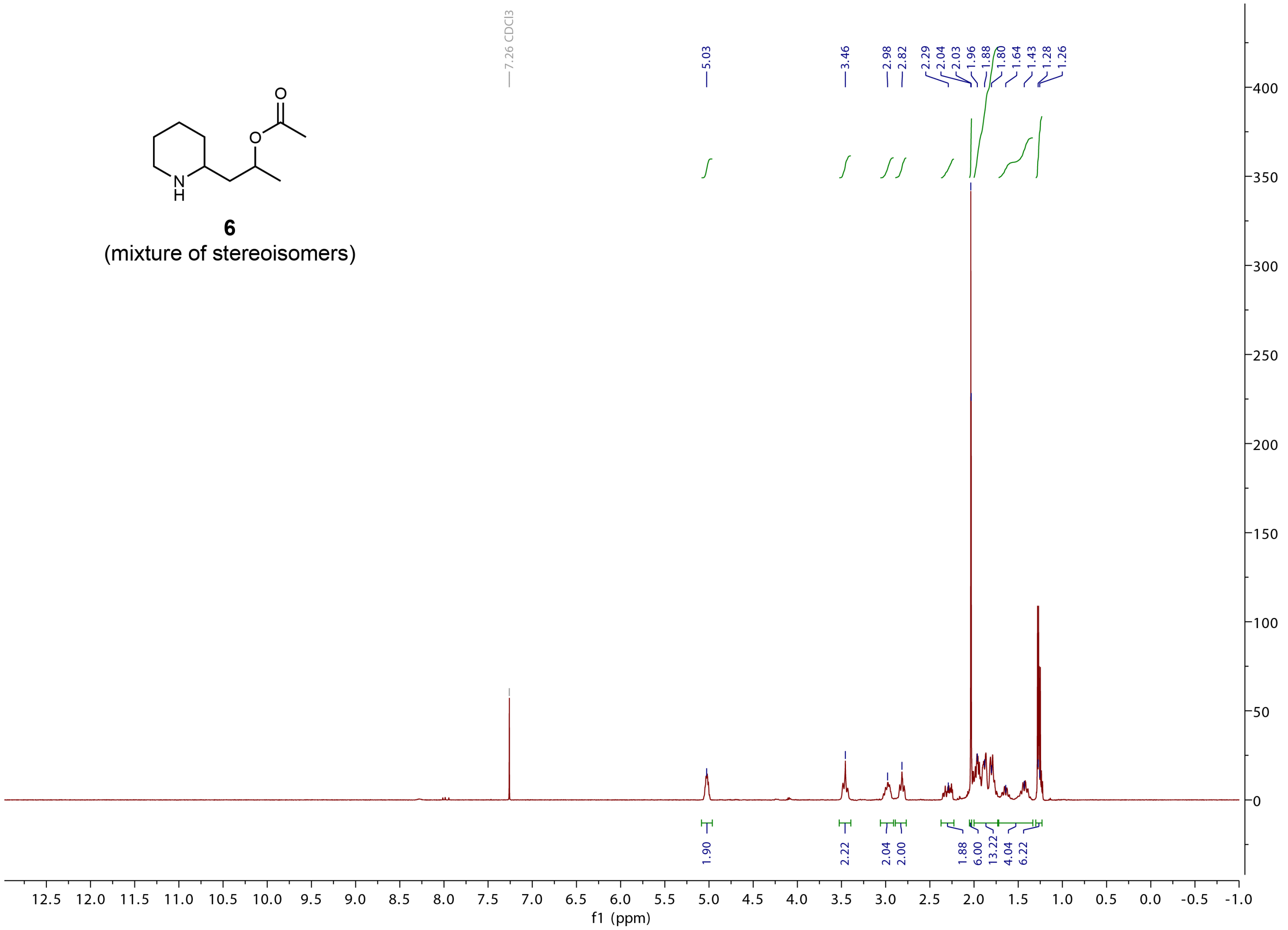

Figure 8. ^{1}H NMR spectrum from the synthesis of 6 stereoisomers. This experiment was recorded at 500 MHz in deuterated chloroform ($CDCl_3$).

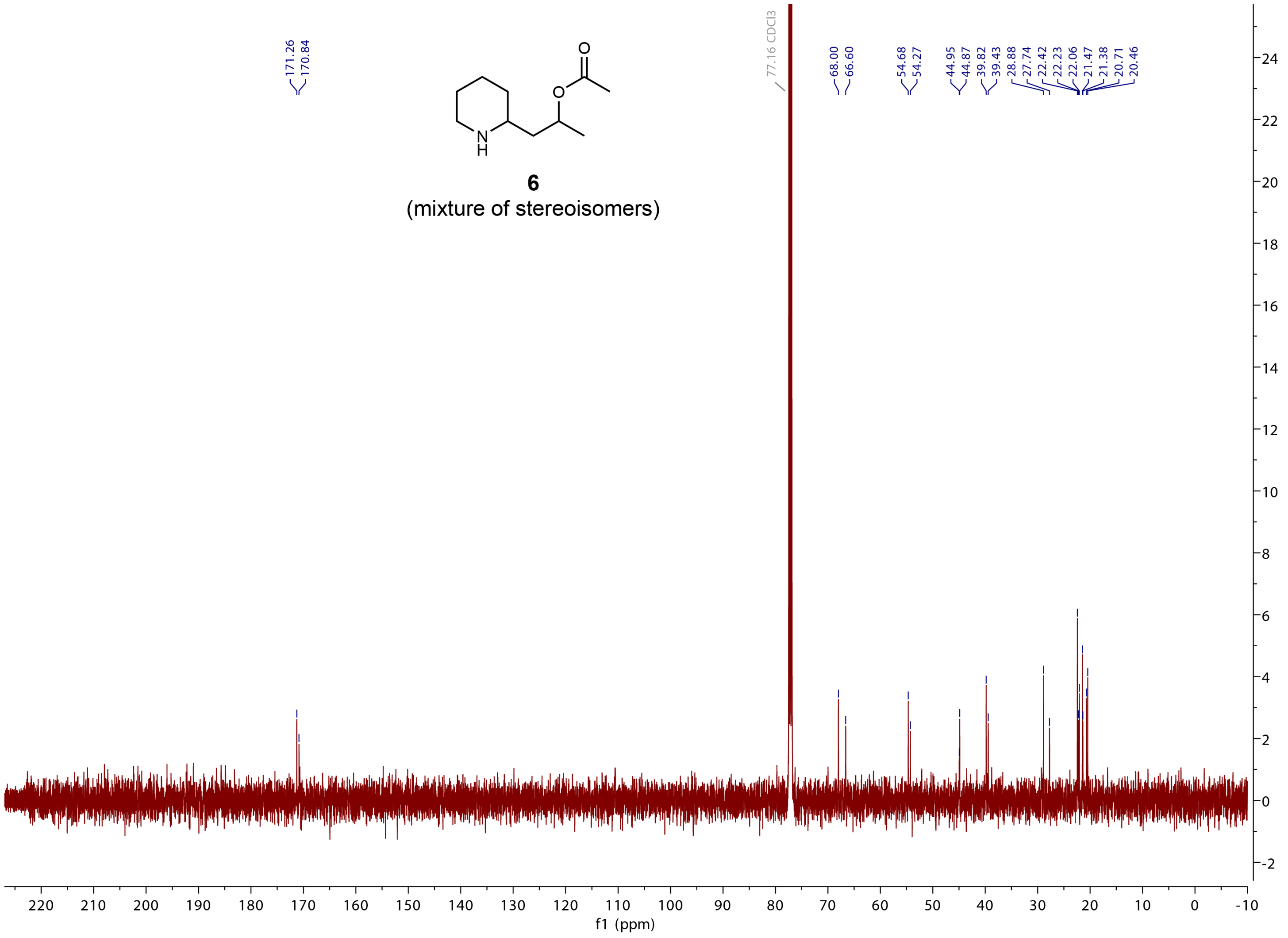

Figure 9. ^{13}C NMR spectrum from the synthesis of 6 stereoisomers. This experiment was recorded at 500 MHz in deuterated chloroform ($CDCl_3$).

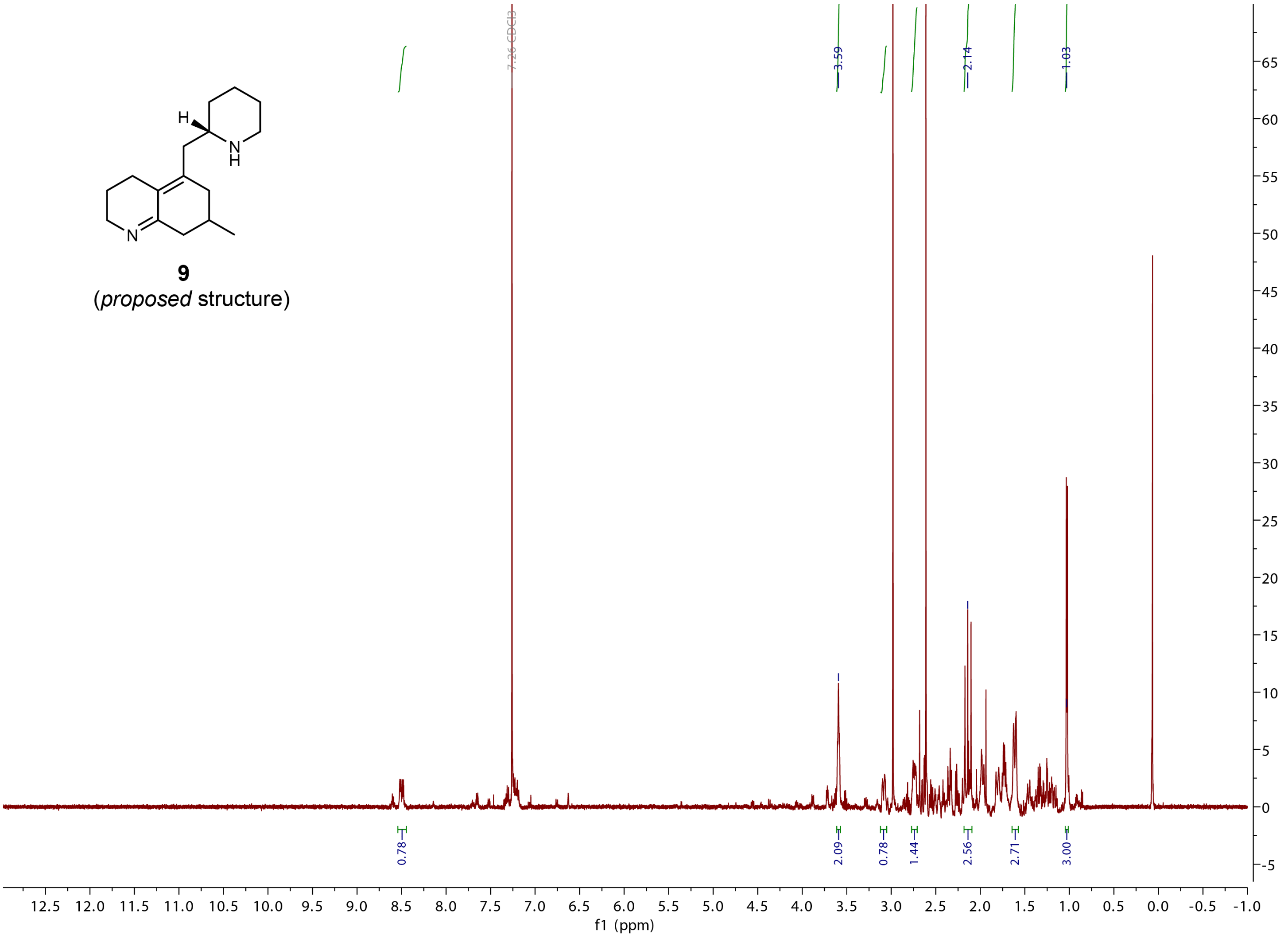

Figure 10. ^{1}H NMR (crude) of the purified product (putative 9, *m/z* 247.2169) of *Pt*CAL-1/*Pt*CAL-2. This experiment was recorded at 500 MHz in deuterated chloroform ($CDCl_3$).

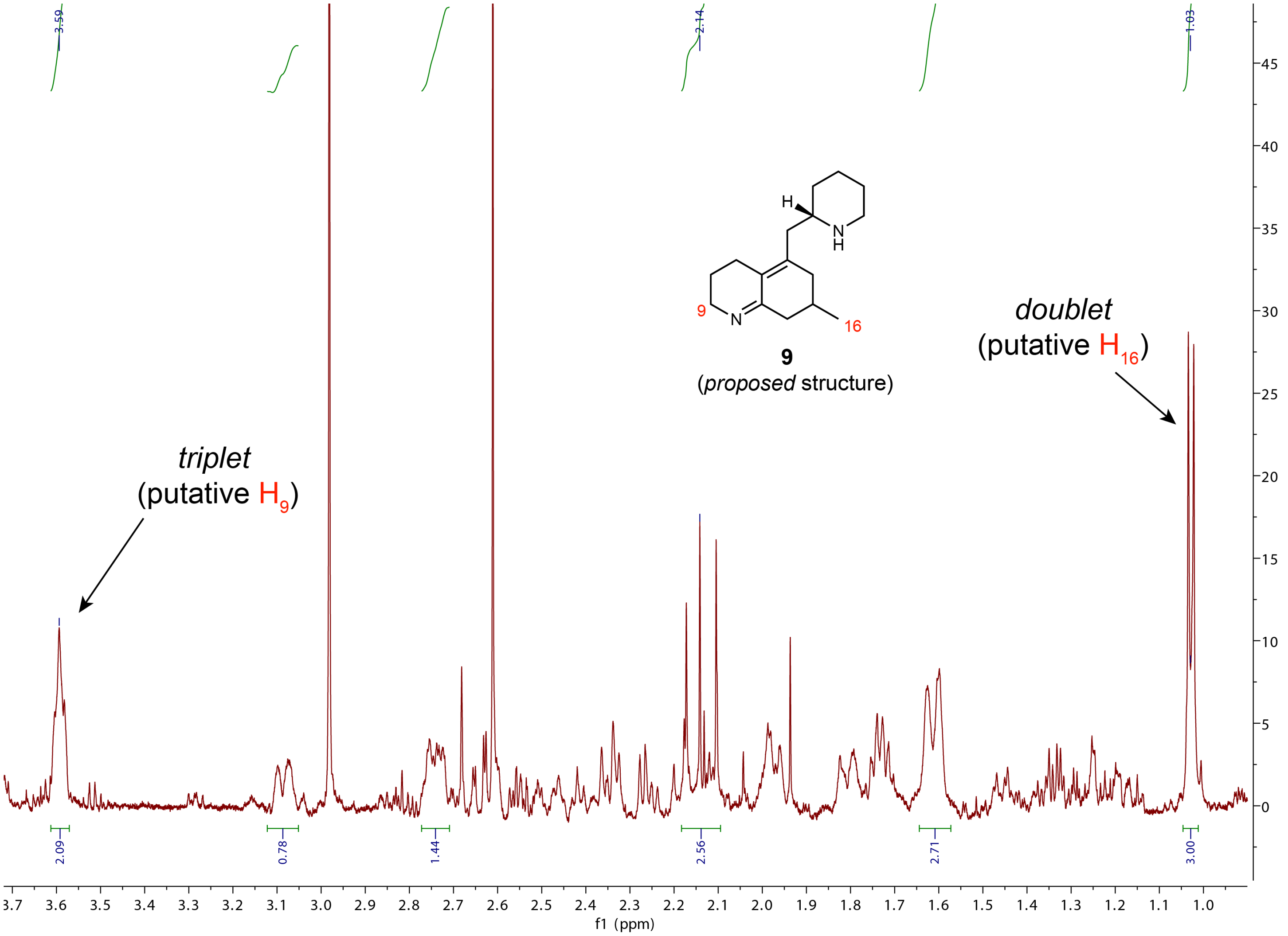

Figure 11. ^{1}H NMR (crude) of putative 9 (zoomed in). Zoomed in view from Figure S10.

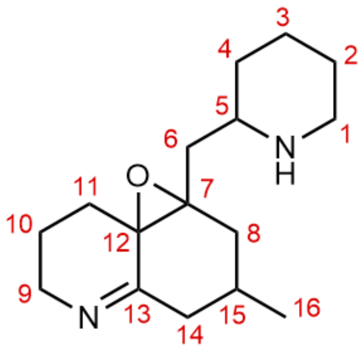

9'
(*proposed*)

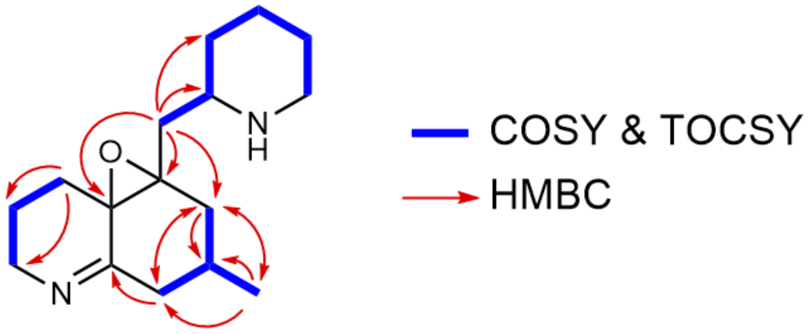

Figure 12. NMR assignment of the oxidized scaffold by-product 9' (*m/z* 263.2118). Assignments, as listed in the table below, are based on the NMR spectra shown in Supplementary Figures 13-18. All experiments were recorded at 600 MHz in deuterated acetonitrile (CD_3CN).

**carbon**		**^{13}C δ (ppm)**	**^{1}H δ (ppm, *J* in Hz)**	
**1**	CH2	47.6	2.58 (1H, td, *J* =11.8, 2.8)	2.97 (1H, dm, *J* = 12.0)
**2**	CH2	27.0	1.32b (1H, m)	1.52 (1H, m)
**3**	CH2	25.7	1.32a (1H, m)	1.74 (1H, m)
**4**	CH2	34.0	1.07 (1H, m)	1.57 (1H, m, *J* = 5.6)
**5**	CH	55.0	2.76 (dddd, *J* = 2.58, 5,46, 8.00, 10.59)	
**6**	CH2	39.9[a]	1.56 (1H, dd, *J* = 5.6, 14,1)	1.65 (1H, dd, *J* = 7.8, 14,1)
**7**	C	66.8[a]	--	
**8**	CH2	38.2	1.51 (1H, dd, *J* = 10.2, 14.5)	2.14 (1H)[b]
**9**	CH2	50.1	3.39 (1H, m)	3.78 (1H, dm, *J* = 17.9)
**10**	CH2	22.1	1.73 (1H, m)	1.77 (1H, m)
**11**	CH2	26.4	1.62 (1H, m)	1.88 (1H, td, *J* = 13.4, 3.9)
**12**	C	57.6[a]	--	
**13**	C	167.2[a]	--	
**14**	CH2	42.7	1.76 (1H, m)	2.28 (1H, d, *J* = 13.8)
**15**	CH	24.8	1.82 (1H, m)	
**16**	CH3	21.5	0.88 (3H, d, *J* = 6.43)	

[a]Annotated based upon HMBC and/or HSQC experiments. [b]Hidden in the 1D proton spectrum.

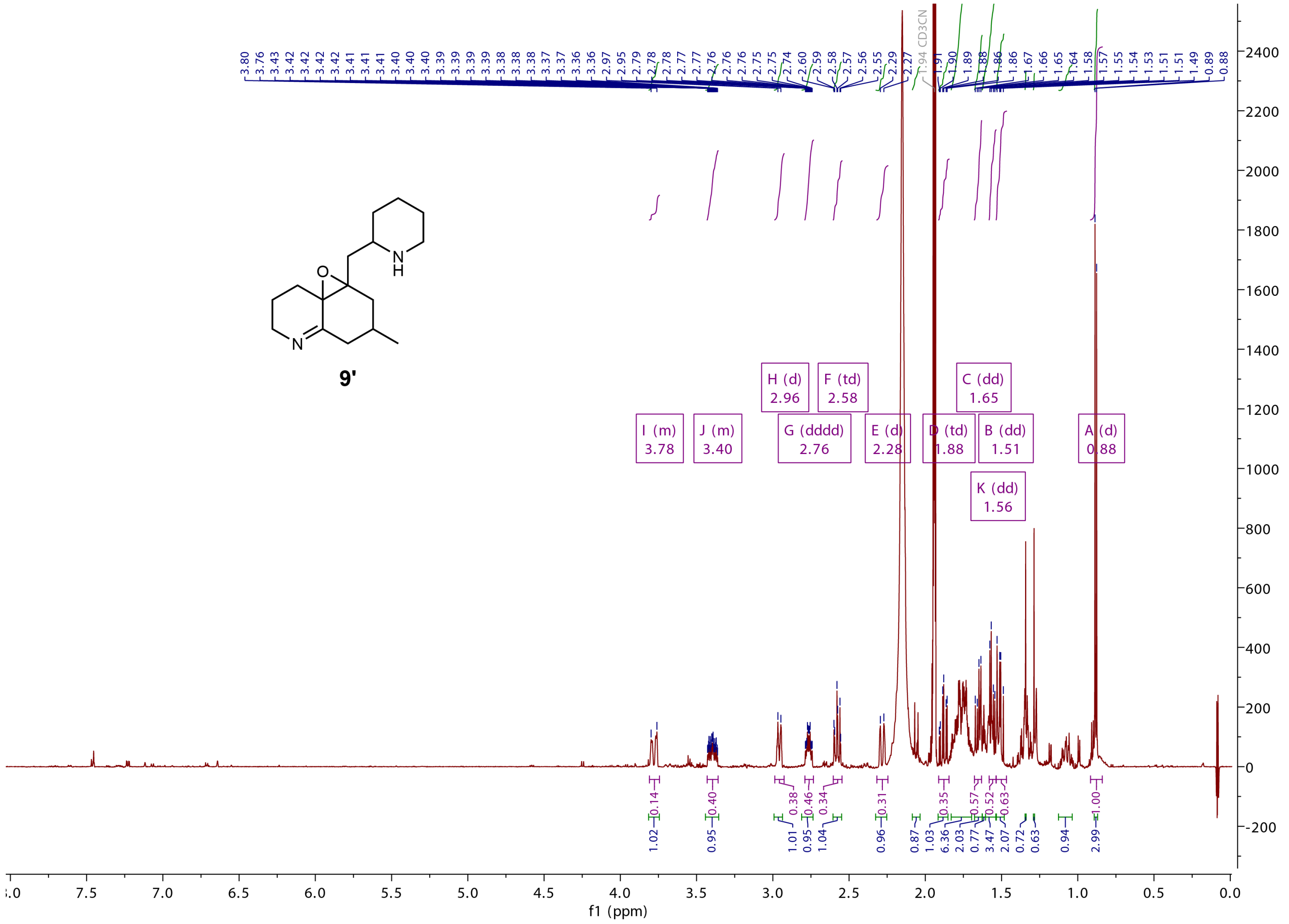

Figure 13. ^{1}H NMR spectrum of the oxidized scaffold by-product 9' (*m/z* 263.2118).

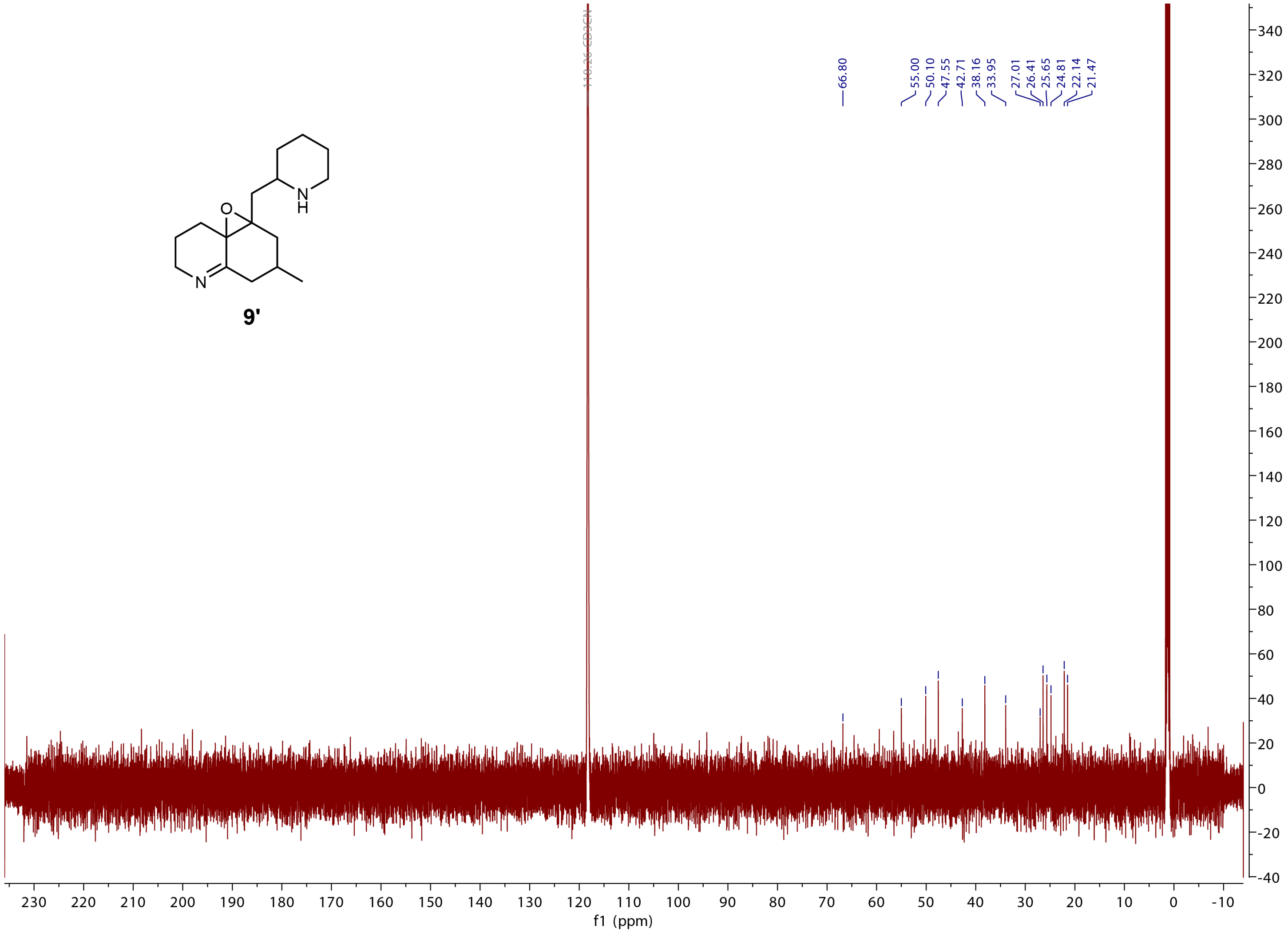

Figure 14. **^{13}C NMR spectrum of the oxidized scaffold by-product 9' (*m/z* 263.2118).**

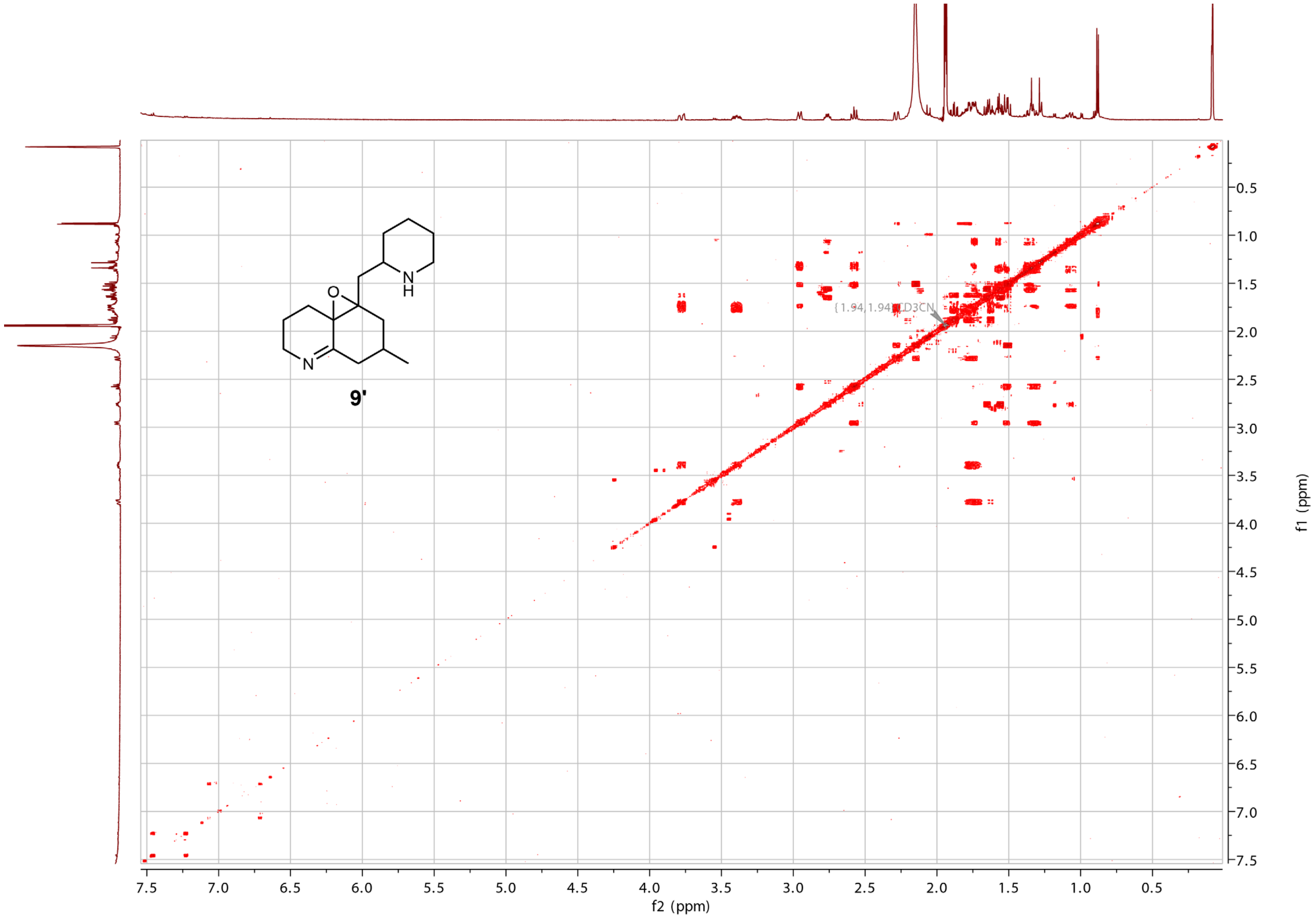

Figure 15. COSY spectrum of the oxidized scaffold by-product 9' (*m/z* 263.2118).

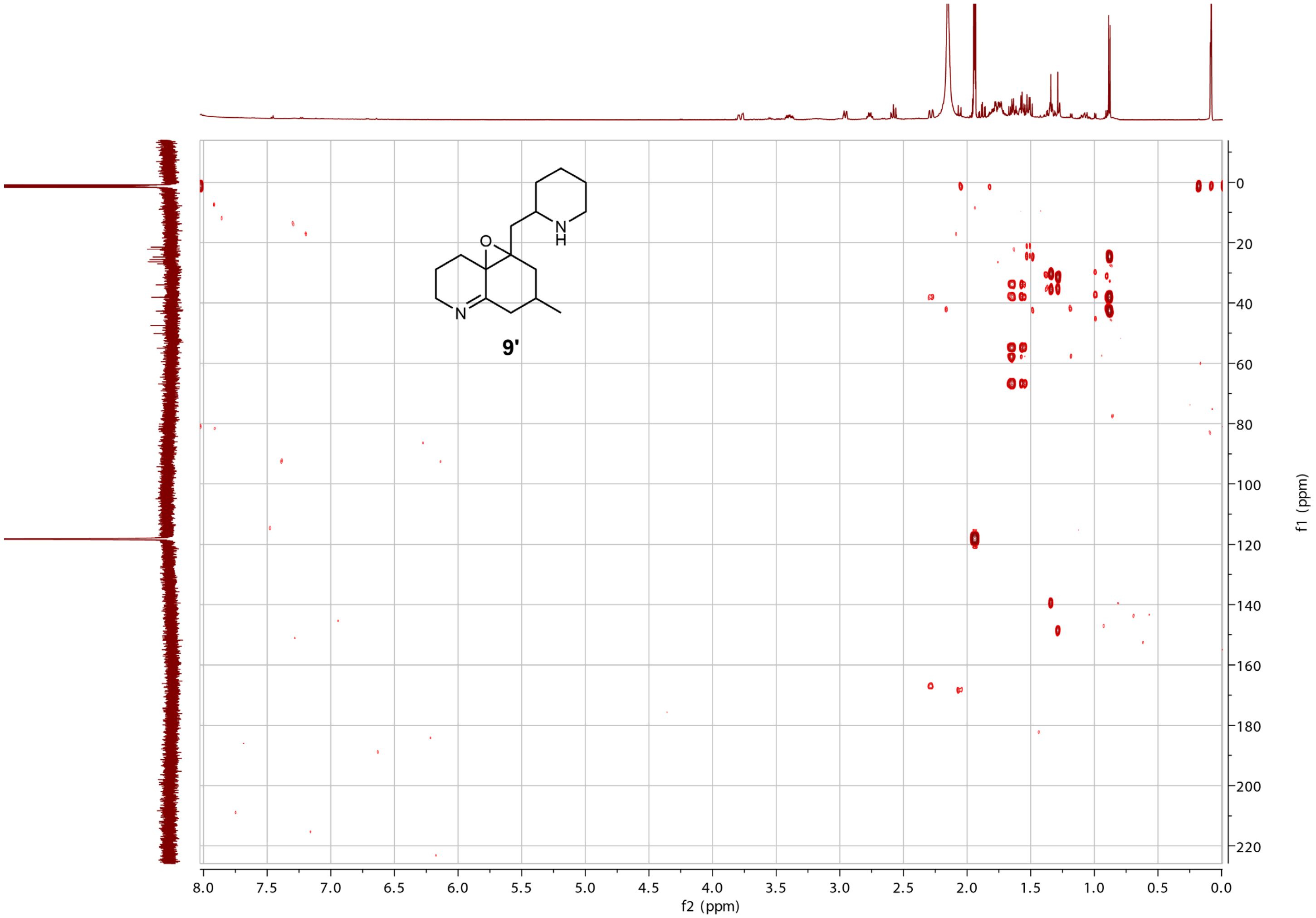

Figure 16. HMBC spectrum of the oxidized scaffold by-product 9' (*m/z* 263.2118).

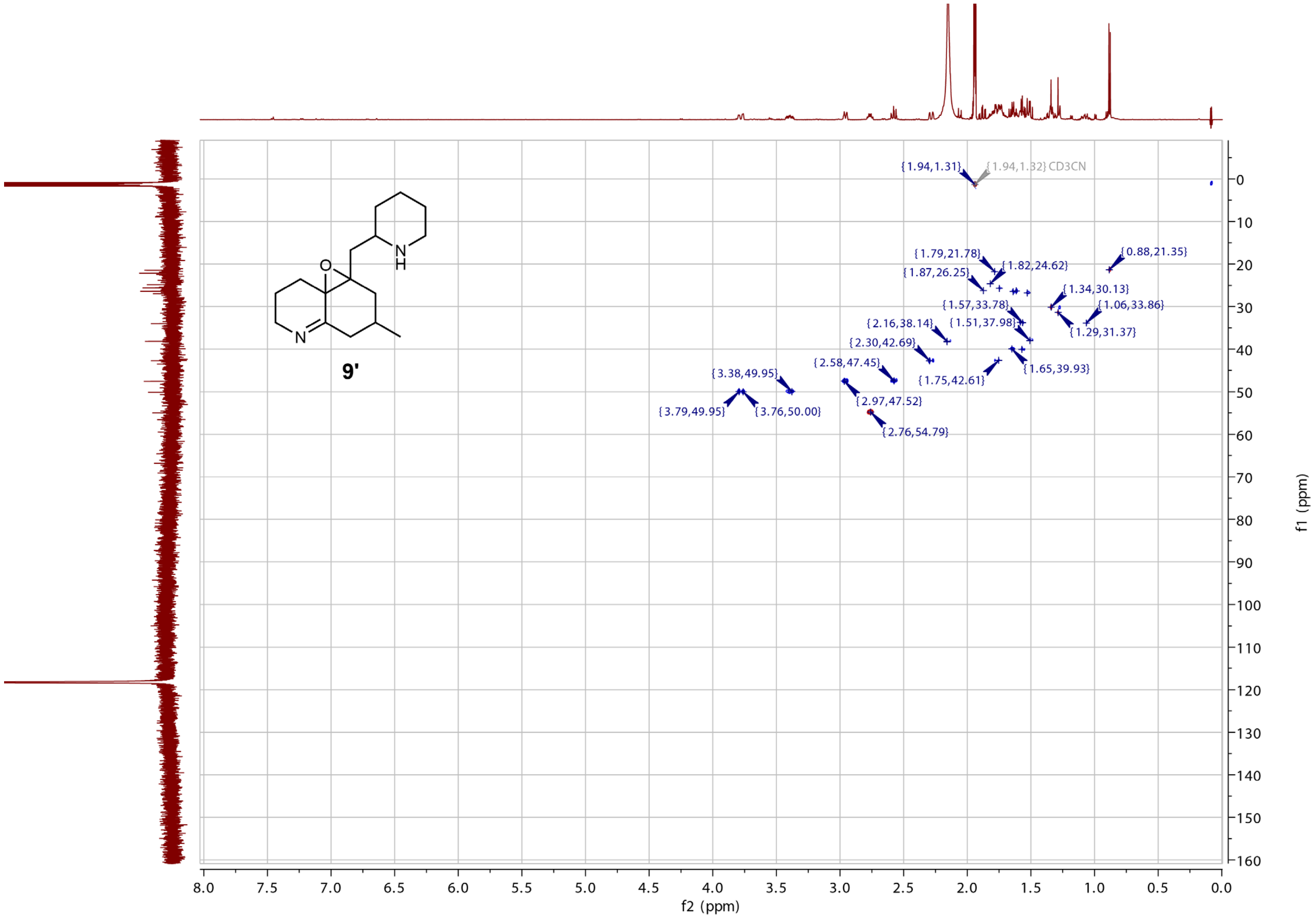

Figure 17. HSQC spectrum of the oxidized scaffold by-product 9' (*m/z* 263.2118).

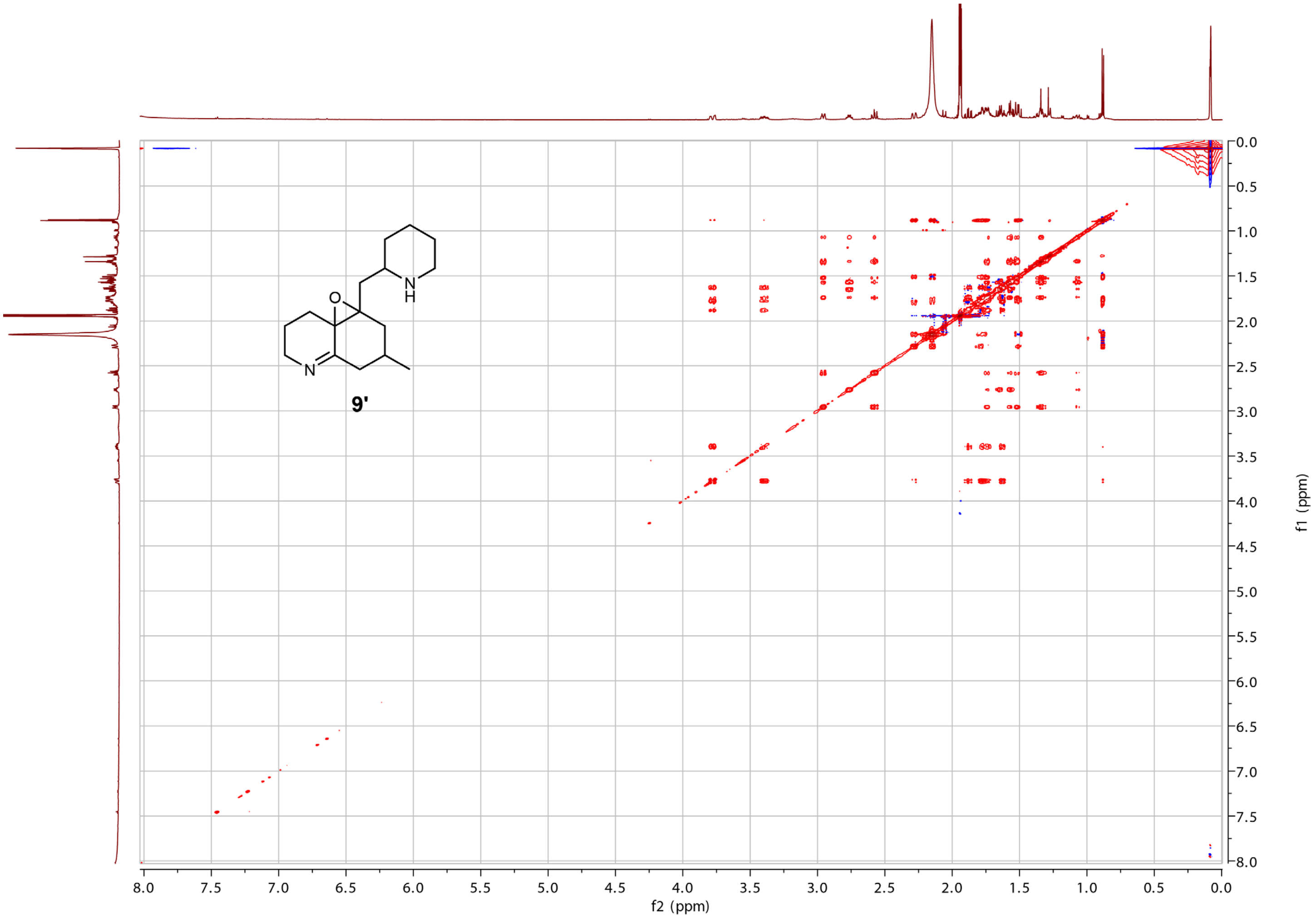

Figure 18. TOCSY spectrum of the oxidized scaffold by-product 9’ (*m/z* 263.2118).

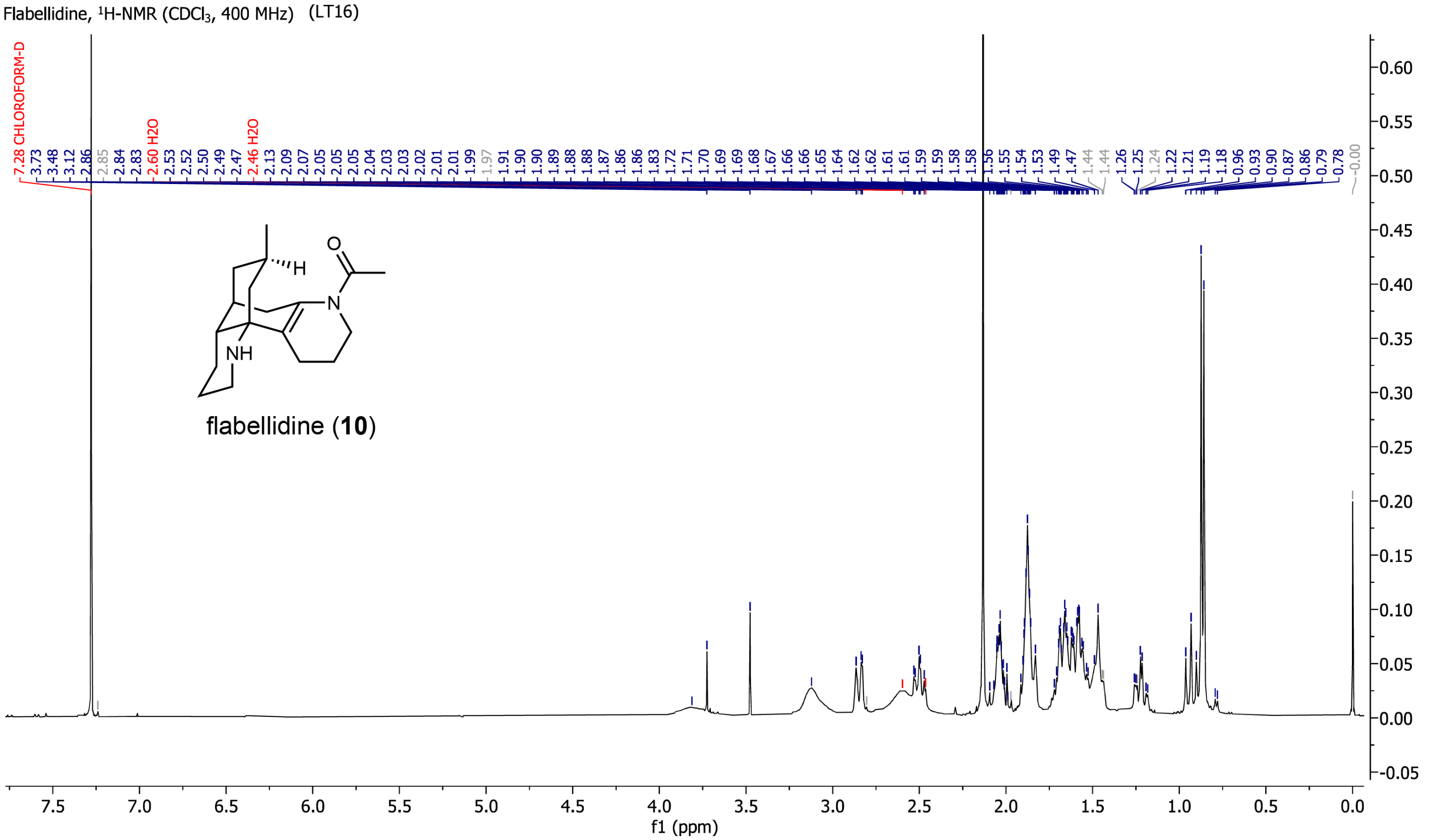

Figure 19. **^{1}H NMR spectrum ($CDCl_3$, 400 MHz) of flabellidine (10) isolated from *Lycopodium platyrhizoma*.**[1]

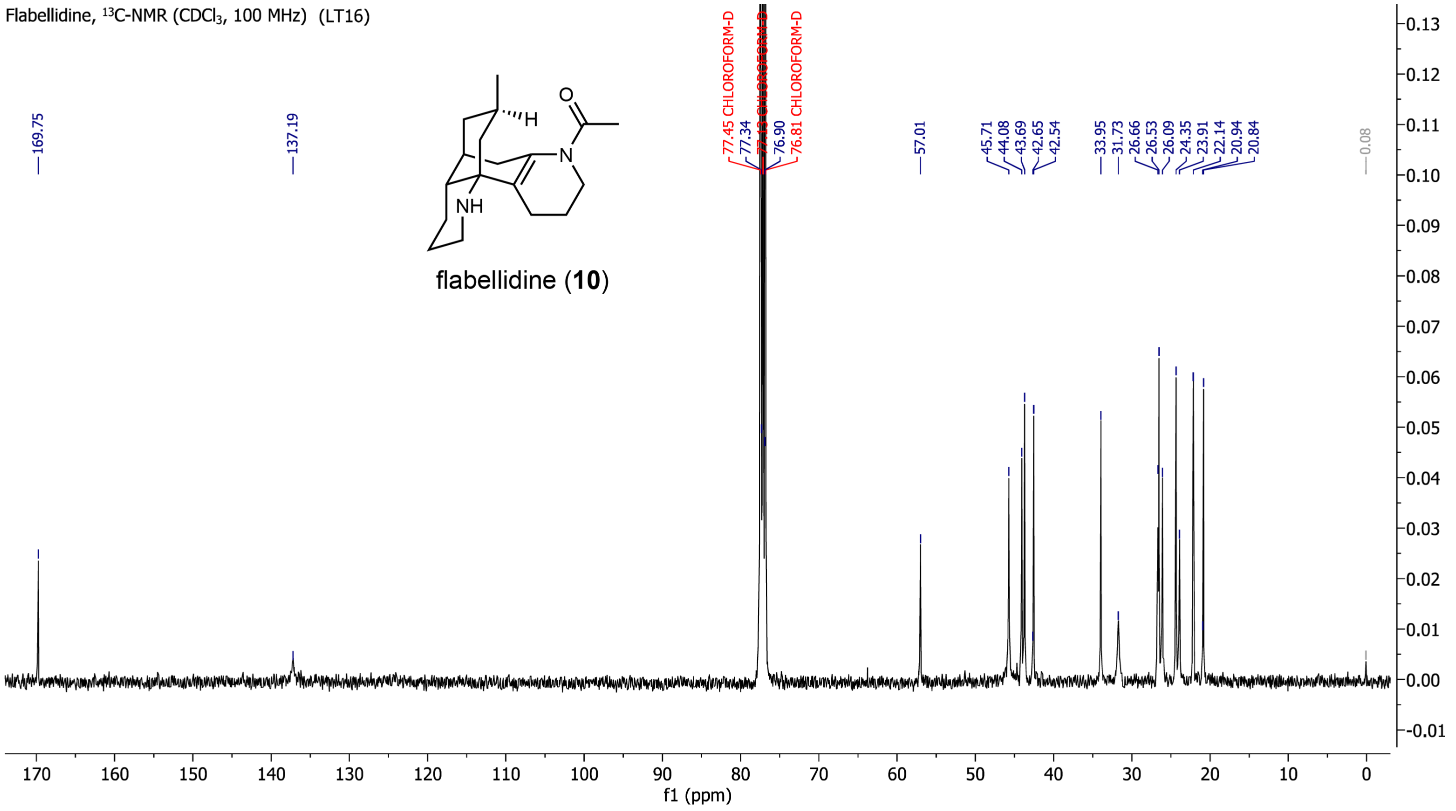

Figure 20. **^{13}C NMR spectrum ($CDCl_3$, 100 MHz) of flabellidine (10) isolated from *Lycopodium platyrhizoma*.**[1]

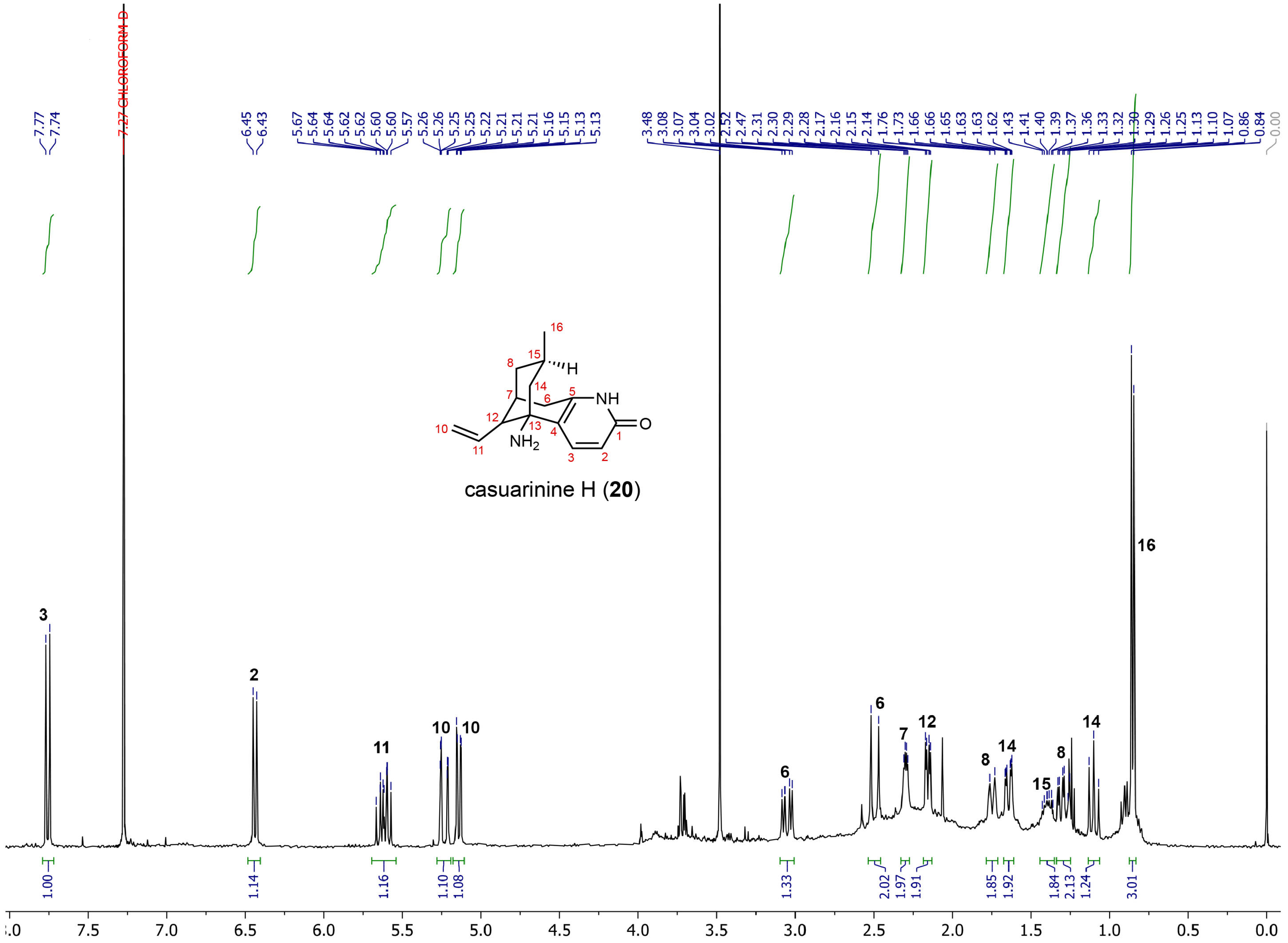

Figure 21. ^{1}**H NMR spectrum (CDCl$_3$, 400 MHz) of casuarinine H (20) isolated from *Lycopodium platyrhizoma*.**[1]

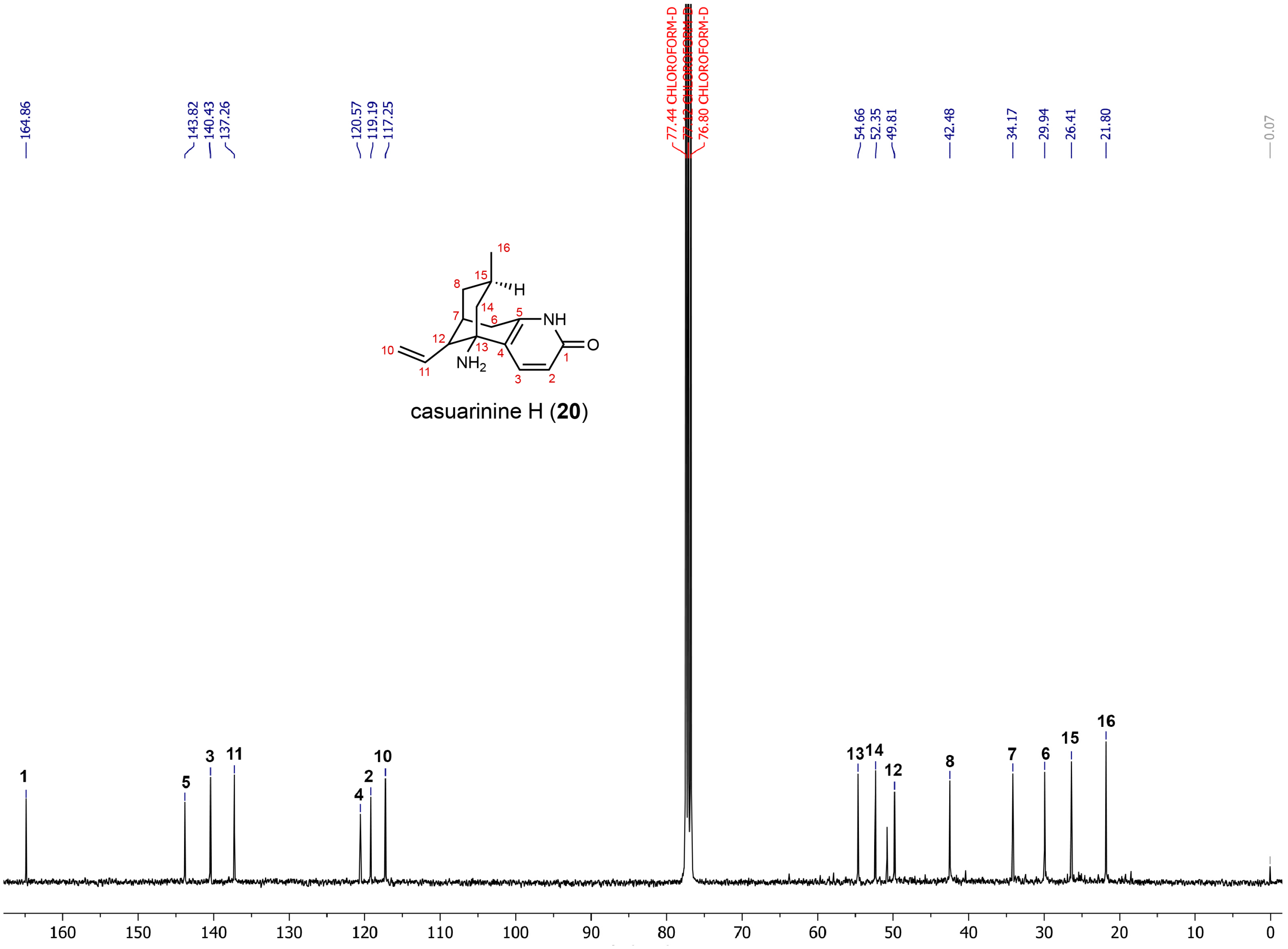

Figure 22. ^{13}**C NMR spectrum (CDCl$_3$, 100 MHz) of casuarinine H (20) isolated from *Lycopodium platyrhizoma*.**[1]

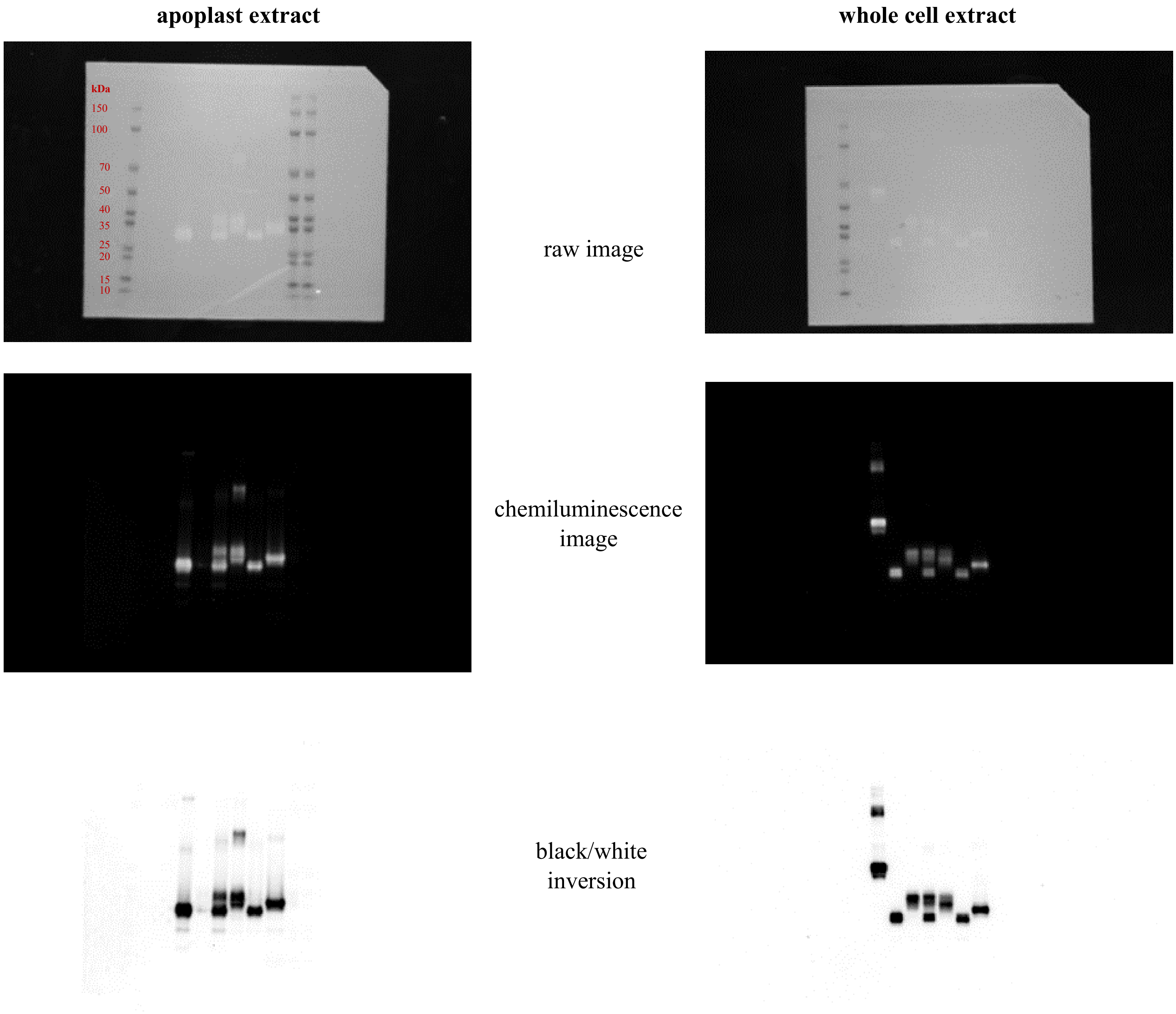

Figure 23. Raw images for Western blots. Images in the left column represent apoplast extract, while those in the right column represent whole cell extract. Top images represent white light images of blots, middle images are chemiluminescence images (taken with an iBright instrument), and the bottom images are the black/while inversions of the chemiluminescence images. To label the protein ladder for the blots shown in **Fig 3b**, chemiluminescence and membrane images were overlaid. Additionally, blots were rotated slightly to ensure straight presentation of lanes. There was no alteration to the chemiluminescence signals.

REFERENCES

1. Yeap, J. S. Y. *et al. Lycopodium* alkaloids: Lycoplatyrine A, an unusual lycodine-piperidine adduct from *Lycopodium platyrhizoma* and the absolute configurations of lycoplanine D and lycogladine H. *J. Nat. Prod.* **82**, 324–329 (2019).

2. Alam, S. N., Adams, A. H. & MacLean, D. B. Lycopodium alkaloids. XV. Structure and mass spectra of some minor alkaloids of *L. flabelliforme*. *Can. J. Chem.* **42**, 2456–2466 (1964).

3. Castillo, M., Gupta, R. N., Ho, Y. K., MacLean, D. B. & Spenser, I. D. Biosynthesis of lycopodine. Incorporation of 1-piperideine and of pelletierine. *Can. J. Bot.* **48**, 2911–2918 (1970).

4. Nett, R. S., Dho, Y., Low, Y.-Y. & Sattely, E. S. A metabolic regulon reveals early and late acting enzymes in neuroactive Lycopodium alkaloid biosynthesis. *Proc. Natl. Acad. Sci.* **118**, e2102949118 (2021).
