# Supplementary material for: Plant carbonic anhydrase-like enzymes in neuroactive alkaloid biosynthesis
Source: Nature. 2023 Nov 8;624(7990):182–91. doi: 10.1038/s41586-023-06716-y (PMC10700139; doi:10.1038/s41586-023-06716-y)
Supplement: Supplementary file 2 — Reporting Summary [file 41586_2023_6716_MOESM2_ESM.pdf]

nature portfolio

Corresponding author(s): Ryan S. Nett & Elizabeth S. Sattely

Last updated by author(s): Sep 15, 2023

# Reporting Summary

Nature Portfolio wishes to improve the reproducibility of the work that we publish. This form provides structure for consistency and transparency in reporting. For further information on Nature Portfolio policies, see our Editorial Policies and the Editorial Policy Checklist.

## Statistics

For all statistical analyses, confirm that the following items are present in the figure legend, table legend, main text, or Methods section.

| n/a | Confirmed | |
|---|---|---|
| ☐ | ☒ | The exact sample size (*n*) for each experimental group/condition, given as a discrete number and unit of measurement |
| ☐ | ☒ | A statement on whether measurements were taken from distinct samples or whether the same sample was measured repeatedly |
| ☐ | ☒ | The statistical test(s) used AND whether they are one- or two-sided<br>*Only common tests should be described solely by name; describe more complex techniques in the Methods section.* |
| ☒ | ☐ | A description of all covariates tested |
| ☐ | ☒ | A description of any assumptions or corrections, such as tests of normality and adjustment for multiple comparisons |
| ☐ | ☒ | A full description of the statistical parameters including central tendency (e.g. means) or other basic estimates (e.g. regression coefficient) AND variation (e.g. standard deviation) or associated estimates of uncertainty (e.g. confidence intervals) |
| ☐ | ☒ | For null hypothesis testing, the test statistic (e.g. *F*, *t*, *r*) with confidence intervals, effect sizes, degrees of freedom and *P* value noted<br>*Give P values as exact values whenever suitable.* |
| ☒ | ☐ | For Bayesian analysis, information on the choice of priors and Markov chain Monte Carlo settings |
| ☒ | ☐ | For hierarchical and complex designs, identification of the appropriate level for tests and full reporting of outcomes |
| ☐ | ☒ | Estimates of effect sizes (e.g. Cohen's *d*, Pearson's *r*), indicating how they were calculated |

*Our web collection on statistics for biologists contains articles on many of the points above.*

## Software and code

Policy information about availability of computer code

| | |
|---|---|
| Data collection | No code was utilized to collect data in this study. |
| Data analysis | Routine data compilation was performed in Microsoft Excel 2016. General analysis of LC-MS data was performed with Agilent MassHunter Qualitative Analysis 10.0. Chromatograms and mass spectra were plotted using IGOR Pro 6.0. Bar graphs and line graphs were plotted using GraphPad Prism 9, and this software was also used for routine statistical analysis. Hierarchical clustering analysis was performed using Cluster 3.0.56 R (version 4.2.2) was used for bar graph generation, visualization of hierarchical clustering data, and for performing XCMS analysis.69 Geneious Prime (version 2019.2.3) was used for bioinformatic analyses of nucleic acid and protein sequences. This software was also used for multiple sequence alignments (MUSCLE algorithm) and phylogenetic tree generation (Jukes-Cantor genetic distance model, Neighbor-Joining tree build method). The TargetP-2.0 server (https://services.healthtech.dtu.dk/service.php?TargetP-2.0)58 was used for predicting signal peptides and protein localization. MNova (v1.6) was used for visualization and processing of NMR data. ChemDraw Professional (version 21.0.0.28) was used for chemical structure visualization and analysis. Structural modeling was performed using AlphaFold-Multimer via ColabFold (v1.5.2), and protein models were visualized in PyMol (version 2.5.4). |

For manuscripts utilizing custom algorithms or software that are central to the research but not yet described in published literature, software must be made available to editors and reviewers. We strongly encourage code deposition in a community repository (e.g. GitHub). See the Nature Portfolio guidelines for submitting code & software for further information.

## Data

Policy information about availability of data

All manuscripts must include a data availability statement. This statement should provide the following information, where applicable:

- Accession codes, unique identifiers, or web links for publicly available datasets
- A description of any restrictions on data availability
- For clinical datasets or third party data, please ensure that the statement adheres to our policy

All data in this manuscript are available upon request. The raw RNA-seq data analyzed in this manuscript have previously been deposited to the NCBI Sequence Read Archive (BioProject PRJNA731132).23 Gene sequences for enzymes characterized in this study are deposited in the National Center for Biotechnology (NCBI) GenBank under the following accessions: Pt2OGD-4 (OR538095), Pt2OGD-5 (OR538096), PtABH-1 (OR538097), PtACT-1 (OR538098), PtCAL-1a (OR538099), PtCAL-1b (OR538100), PtCAL-2a (OR538101), PtCAL-2b (OR538102), PtCAL-3 (OR538103), PtCYP782C1 (four homologs; OR538104, OR538105, OR538106, OR538107), PtSDR-1 (OR538108), and PtSDR-2 (OR538109). The UniProt database (https://www.uniprot.org/) was used for identifying and obtaining CAH family sequences that were used in phylogenetic analyses. The human CA2 protein structure (2vva) was acquired from PDB (https://www.rcsb.org/). Any materials generated within this manuscript will be made available, as possible.

## Research involving human participants, their data, or biological material

Policy information about studies with human participants or human data. See also policy information about sex, gender (identity/presentation), and sexual orientation and race, ethnicity and racism.

| | |
|---|---|
| Reporting on sex and gender | N/A |
| Reporting on race, ethnicity, or other socially relevant groupings | N/A |
| Population characteristics | N/A |
| Recruitment | N/A |
| Ethics oversight | N/A |

Note that full information on the approval of the study protocol must also be provided in the manuscript.

# Field-specific reporting

Please select the one below that is the best fit for your research. If you are not sure, read the appropriate sections before making your selection.

☒ Life sciences ☐ Behavioural & social sciences ☐ Ecological, evolutionary & environmental sciences

For a reference copy of the document with all sections, see nature.com/documents/nr-reporting-summary-flat.pdf

# Life sciences study design

All studies must disclose on these points even when the disclosure is negative.

| | |
|---|---|
| Sample size | In the experiments within this manuscript, we typically used a sample size of three replicates in order to have the power for statistical comparisons. |
| Data exclusions | No data has been excluded from any of the statistical analyses. |
| Replication | All experiments were replicated at least once, and in most circumstances, in greater than three independent experiments. |
| Randomization | Randomization is not relevant to the experiments of this manuscript. The various experimental conditions were specifically defined to probe for the function of distinct enzymes, and there was no random assigning of samples to experiment groups. |
| Blinding | We were not blinded to our analysis; experiments were designed to probe for the function of individual enzymes, and this required the researchers to know which samples contained which combination of enzymes. Thus, in general, blinding was not relevant to our studies. |

# Reporting for specific materials, systems and methods

We require information from authors about some types of materials, experimental systems and methods used in many studies. Here, indicate whether each material, system or method listed is relevant to your study. If you are not sure if a list item applies to your research, read the appropriate section before selecting a response.

## Materials & experimental systems

| n/a | Involved in the study |
|---|---|
| ☐ | ☒ Antibodies |
| ☒ | ☐ Eukaryotic cell lines |
| ☒ | ☐ Palaeontology and archaeology |
| ☒ | ☐ Animals and other organisms |
| ☒ | ☐ Clinical data |
| ☒ | ☐ Dual use research of concern |
| ☐ | ☒ Plants |

## Methods

| n/a | Involved in the study |
|---|---|
| ☒ | ☐ ChIP-seq |
| ☒ | ☐ Flow cytometry |
| ☒ | ☐ MRI-based neuroimaging |

# Antibodies

Antibodies used

1. Mouse anti-His antibody antibody (Genscript A00186) for detecting the presence of His-tagged proteins in our Nicotiana benthamiana gene expression system.
2. Horse anti-mouse IgG, HRP-linked antibody (Cell signaling Technology 7076) for Western blots.

Validation

1. The mouse anti-His antibody antibody (Genscript A00186) is validated by the manufacturer to be specific to 6x His-tagged proteins via ELISA. This antibody has been shown to also be effective against 4x and 5x His tags and to work with both native and denatured synthetic proteins produced in diverse heterologous systems. Further information, including certificates of analysis and additional information on validation are available on the manufacturer website. (https://www.genscript.com/antibody/A00186-THE_His_Tag_Antibody_mAb_Mouse.html).
2. The horse anti-mouse IgG, HRP-linked antibody (Cell signaling Technology 7076) is thoroughly validated by the manufacturer with Cell Signaling Technology primary antibodies. Additional information, including certificates of analysis and antibody validation are available on the manufacturer website. (https://www.cellsignal.com/products/secondary-antibodies/anti-mouse-igg-hrp-linked-antibody/7076?_requestid=19655)

# Dual use research of concern

Policy information about dual use research of concern

## Hazards

Could the accidental, deliberate or reckless misuse of agents or technologies generated in the work, or the application of information presented in the manuscript, pose a threat to:

| No | Yes |
|---|---|
| ☒ | ☐ Public health |
| ☒ | ☐ National security |
| ☒ | ☐ Crops and/or livestock |
| ☒ | ☐ Ecosystems |
| ☒ | ☐ Any other significant area |

## Experiments of concern

Does the work involve any of these experiments of concern:

| No | Yes |
|---|---|
| ☒ | ☐ Demonstrate how to render a vaccine ineffective |
| ☒ | ☐ Confer resistance to therapeutically useful antibiotics or antiviral agents |
| ☒ | ☐ Enhance the virulence of a pathogen or render a nonpathogen virulent |
| ☒ | ☐ Increase transmissibility of a pathogen |
| ☒ | ☐ Alter the host range of a pathogen |
| ☒ | ☐ Enable evasion of diagnostic/detection modalities |
| ☒ | ☐ Enable the weaponization of a biological agent or toxin |
| ☒ | ☐ Any other potentially harmful combination of experiments and agents |
